# 12b,24b‐Diborahexabenzo[*a*,*c*,*fg*,*l*,*n*,*qr*]pentacene: A Low‐LUMO Boron‐Doped Polycyclic Aromatic Hydrocarbon

**DOI:** 10.1002/anie.202115746

**Published:** 2022-01-03

**Authors:** Carina Mützel, Jeffrey M. Farrell, Kazutaka Shoyama, Frank Würthner

**Affiliations:** ^1^ Institut für Organische Chemie Universität Würzburg Am Hubland 97074 Würzburg Germany; ^2^ Center for Nanosystems Chemistry (CNC) Universität Würzburg Theodor-Boveri-Weg 97074 Würzburg Germany

**Keywords:** Aromaticity, Boron, Near infrared emitter, Pentacene, Polycycles

## Abstract

*Herein we devise and execute a new synthesis of a pristine boron‐doped nanographene. Our target boron‐doped nanographene was designed based on DFT calculations to possess a low LUMO energy level and a narrow band gap derived from its precise geometry and B‐doping arrangement. Our synthesis of this target, a doubly B‐doped hexabenzopentacene (**B***_***2***_ ***‐HBP**), employs six net C−H borylations of an alkene, comprising consecutive hydroboration/electrophilic borylation/dehydrogenation and BBr_3_/AlCl_3_/2,6‐dichloropyridine‐mediated C−H borylation steps. As predicted by our calculations, **B***_***2***_ ***‐HBP** absorbs strongly in the visible region and emits in the NIR up to 1150* *nm in o‐dichlorobenzene solutions. Furthermore, **B***_***2***_ ***‐HBP** possesses a very low LUMO level, showing two reversible reductions at −1.00* *V and −1.17* *V vs. Fc^+^/Fc. Our methodology is surprisingly selective despite its implementation of unfunctionalized precursors and offers a new approach to the synthesis of pristine B‐doped polycyclic aromatic hydrocarbons*.

## Graphical abstract

A doubly B‐doped hexabenzopentacene was designed based on presynthetic DFT calculations, predicting intriguing optoelectronic properties. Subsequently, the electron‐deficient, NIR‐emitting B‐doped polycyclic aromatic hydrocarbon was prepared via six net C−H borylations from an unfunctionalized alkene in a two‐pot synthetic procedure. The structure obtained by X‐ray crystallography confirmed an enhanced π‐conjugation upon cyclization compared to non‐fused derivatives.

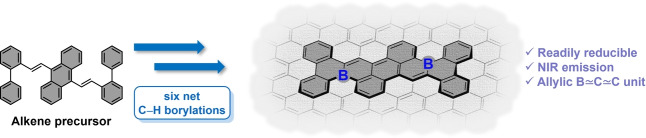

Polycyclic aromatic hydrocarbons (PAHs) are fundamentally fascinating structures as both functional materials and as substructures of larger graphitic molecules.^[1]^ Nevertheless, the study of “nanographenes” confines structural space to hexagonal arrays of sp^2^ carbon. This stipulation presents two challenges. First, properties may only be adjusted using different arrangements of six‐membered rings, edge functionalization or by embedding heteroatoms (doping).^[2]^ Second, the syntheses of atom‐precise PAHs are demanding. Specifically, boron‐doped PAHs are often challenging to synthesize, but are intriguing as electron‐deficient functional organic materials.^[3]^ Particularly appealing are PAHs with precise boron substitutions at non‐edge positions; these may benefit from stability‐enhancing “structural constraint” at boron.^[4]^ However, few examples of these compounds are reported.[^4^, ^5^] Seminal examples include doubly B‐doped nanographene **I** from Yamaguchi and co‐workers,^[5a]^ and recently reported tetrabenzopentacene **IV** from Wagner and co‐workers^[5c]^ (Figure 1). Both nanographenes are remarkable examples and show interesting properties, such as strong broad absorption in the UV/Visible range for **I** or strong blue emission for **IV**, as well as sufficient Lewis acidity to bind anions or Lewis bases. Unfortunately, the limited range of synthetic strategies for variations of **I** and **IV** hampers further optimization of electronic properties for organic electronics.

**Figure 1:**
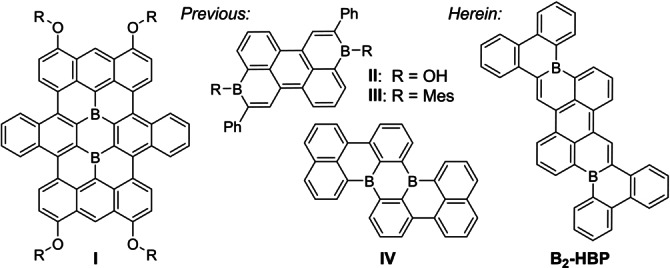
Recently reported examples of doubly boron‐doped PAHs **I** to **IV** and new compound **B_2_‐HBP** reported herein.

In recent work, we developed a general C−H borylation synthesis of boron‐doped PAHs including **II** and **III** and found that DFT calculations could predict how size, shape, and arrangement of boron atoms in the π‐core affect optoelectronic properties.^[6]^ Herein, we use DFT calculations to design a boron‐doped PAH possessing a low LUMO energy level and a narrow band gap where boron atoms are embedded in non‐edge positions. We synthesize this fully fused boron‐doped PAH using our C−H borylation protocol for boron‐doped PAHs in combination with Lewis acid/base mediated C−H borylation of pendant biphenyl groups. Our spectroscopic and voltammetric measurements of **B_2_‐HBP** reveal a low‐LUMO, long wavelength absorbing, and deep‐red NIR emitting material in line with our predictions. This illustrates the effectiveness of DFT‐calculated design and C−H borylation synthesis as a combined approach to pristine B‐doped nanocarbons.

We set out to synthesize a new, large, and fully fused boron‐doped nanographene with a low LUMO level and intense absorption at the NIR edge of the visible range as desired for a variety of applications (e.g. as an n‐type semiconductor for transistors or solar cells, or as a near‐infrared absorbing or emitting material).^[7]^ The core arrangement of **III** was chosen as a starting point, as it showed the lowest energy absorption band and lowest calculated LUMO level amongst a series of B‐doped PAHs recently reported by our group.^[6b]^ We envisioned that a large, “fully fused” compound based on **III** would provide access to an easy reducible B‐doped PAH exhibiting π–π interactions, and long wavelength absorption and emission due to its extended π‐system. Large acenes such as pentacene and their derivatives have drawn great interest in the past decades as materials for organic electronics.[^7a^, ^7b^, ^8^]

Nevertheless, noting the stark electronic effects of core variations in our previous series of B‐doped PAHs, we performed DFT calculations on the B3LYP/6‐311G** level of theory for **III** and for newly designed **B_2_‐HBP**, a doubly B‐doped hexabenzopentacene, for which the isostructural all‐carbon analog is hitherto unknown (see Supporting Information, Figures S22–S25). We also calculated the frontier orbitals of the unmethylated derivative of **III** (see Supporting Information, Figures S26, S27) to verify that methyl groups have only negligible effects on the frontier orbitals of **III**. DFT calculations predict a 0.20 eV higher HOMO energy level and a 0.02 eV lower LUMO energy level for **B_2_‐HBP** compared to **III**. Therefore, the frontier molecular orbital energy gap for **B_2_‐HBP** is predicted to be considerably smaller than that of **III** and to result in a bathochromically (≈60 nm) shifted absorption spectrum. DFT calculations also predict effective delocalization of the frontier molecular orbitals over the entire π‐scaffold, including fused aryl residues (see Figure 2). The stronger influence of “planarization” on the HOMO level can be attributed to a higher delocalization of HOMO over the whole molecule including the electron‐rich phenyl substituent for **III**. Conversely, the LUMO is mostly located at the diboraperylene core (see Supporting Information, Figure S25). Therefore annulation of **III** has a higher impact on the HOMO level compared to the LUMO level for **B_2_‐HBP** as has been observed for other donor–acceptor systems.^[9]^ Oscillator strengths simulated by TD‐DFT (B3LYP/6‐311G**) calculations predict stronger absorption of B‐containing **B_2_‐HBP**, with oscillator strengths of 0.589 for HOMO→LUMO transition and 0.140 for HOMO‐2→LUMO compared to **III** with oscillator strengths of 0.509 for HOMO→LUMO transition. With DFT calculations intimating **B_2_‐HBP** as a promising candidate for a low‐LUMO doubly B‐doped PAH, we attempted its synthesis following Scheme 1.

**Figure 2:**
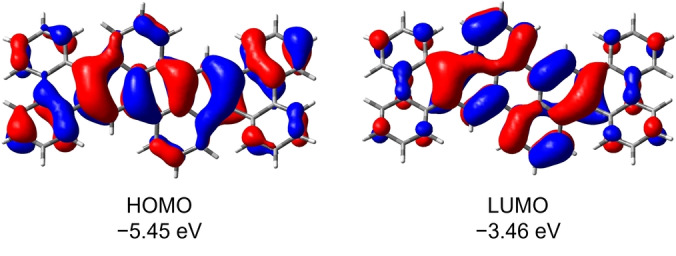
Frontier molecular orbitals of **B_2_‐HBP** from DFT calculations at the B3LYP/6‐311G** level of theory.

**Scheme 1:**
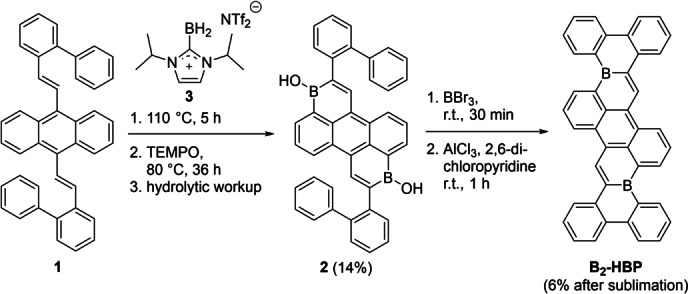
Synthesis of borinic acid **2** and doubly boron‐doped hexabenzopentacene **B_2_‐HBP**.

The alkene precursor for our synthesis (**1**) was synthesized via Horner–Wadsworth–Emmons‐reaction of 9,10‐bis(diethylphosphonomethyl)anthracene and 2‐phenylbenzaldehyde in good yield (80 %). The alkene **1** was reacted with an in situ generated borenium salt **3** in chlorobenzene to give borinic acid **2** in 14 % yield via a hydroboration/C−H borylation/dehydrogenation cascade reaction after treatment with TEMPO radical and hydrolytic workup.^[6a]^ Subsequently, borinic acid **2** was dissolved in dichloromethane, treated with excess BBr_3_ and stirred at room temperature for 30 min to effect OH for Br exchange. Residual BBr_3_ and solvent were removed under reduced pressure and various reaction conditions were tested for the final electrophilic C−H borylations (see Supporting Information, Table S1). No borylations occurred when this dibrominated intermediate was heated at 100 °C or at 125 °C for 16 hours in toluene. Higher temperature reactions (150 °C) yielded apparent decomposition products. Lewis acids (AlCl_3_, FeCl_3_, Sc(OTf)_3_) and various bases (Hünig's base,^[5i]^ LiHMDS or 2,6‐dichloropyridine) were probed to mediate the desired borylation reactions. When used individually, these additives did not furnish **B_2_‐HBP**. However, conversion to the desired doubly boron‐doped nanographene was observed when both a base (2,6‐dichloropyridine or/and 2,6‐di‐*tert*‐butylpyridine), and a Lewis acid (AlCl_3_) were employed in concert, analogous to borylation conditions reported by Ingleson and co‐workers.^[10]^ The reaction was most efficient at room temperature, with decomposition predominating at higher temperature (150 °C). Nanographene **B_2_‐HBP** shows low solubility in most organic solvents indicating strong intermolecular interactions. Nevertheless, spectroscopically pure **B_2_‐HBP** could be isolated in 6 % yield (over two steps from borinic acid **2**) by washing the reaction product with solvent (hexane, toluene, and dichloromethane) and subjecting the resulting residue to gradient sublimation (370 °C, 10^−4^ mbar).

The optical properties of borinic acid **2** and B‐doped PAH **B_2_‐HBP** were studied by UV/Vis absorption and emission spectroscopies in dichloromethane (**2**, **B_2_‐HBP**) and in *o*‐dichlorobenzene (**B_2_‐HBP**) solutions at room temperature (see Figure 3 and Supporting Information, Figures S15, S16). The optical properties of **2** are similar to the previously reported **II**, with which it shares structural similarity.^[6a]^ Thus, borinic acid **2** shows a well resolved S_0_−S_1_ transition at *λ*=560 nm (26 600 M^−1^ cm^−1^) and a mirror‐image fluorescence with a *λ*_max_ of 601 nm corresponding to a Stokes shift of 1220 cm^−1^ (see Supporting Information, Figure S15). The fluorescence quantum yield is *Φ*=0.70 measured via relative method, with a lifetime of 5.51 ns. **B_2_‐HBP** exhibits a similar band shape to **2** where the lowest energy absorption maximum corresponds to the S_0_–S_1_ transition with a well‐resolved vibronic progression. However, this extended π‐scaffold exhibits a strongly bathochromically shifted absorption spectrum with absorption maxima at 692 nm in dichloromethane (*ϵ*_max_=35 900 M^−1^ cm^−1^) and 704 nm in *o*‐dichlorobenzene (*ϵ*_max_=31 000 M^−1^ cm^−1^). If compared to the non‐fused molecule **III**, the experimentally determined bathochromic shift (1870 cm^−1^, 81 nm) is in agreement with the theoretically determined value (1310 cm^−1^, 60 nm). The oscillator strengths simulated by (TD‐)DFT calculations fit very well with the observed extinction coefficients (see Supporting Information, Figure S16). The emission spectrum of **B_2_‐HBP** exhibits mirror‐image fluorescence up to 1150 nm with a maximum at 757 nm. To the best of our knowledge, **B_2_‐HBP** exhibits the longest‐wavelength emission observed in solution for an unsubstituted doubly B‐doped nanographene,[^5a^, ^5c^] and emits mainly in the NIR region.^[11]^ In comparison to tetrabenzopentacene **IV**, exhibiting a very similarly sized π‐scaffold, the difference in the optical properties is surprisingly severe, as the absorption and emission maxima for **B_2_‐HBP** are bathochromically shifted by >200 nm. The resulting Stokes shift is 1000 cm^−1^ and therefore smaller than that of borinic acid **2** (1220 cm^−1^) and structurally related non‐fused **III** (1400 cm^−1^). This is in accordance with other structurally rigidified organoboranes[^3e^, ^5e^] where smaller Stokes shifts are attributed to lesser structural reorganisation on excitation. The relative fluorescence quantum yield of **B_2_‐HBP** was determined as *Φ*= 0.06 with a short lifetime of 1.00 ns. The diminished fluorescence quantum yield compared to **2** and **III** (see Supporting Information, Table S2) is in accordance with the energy‐gap law, in which nonradiative decay becomes more favourable at smaller energy separations and therefore the fluorescence quantum yield decreases.^[12]^

**Figure 3:**
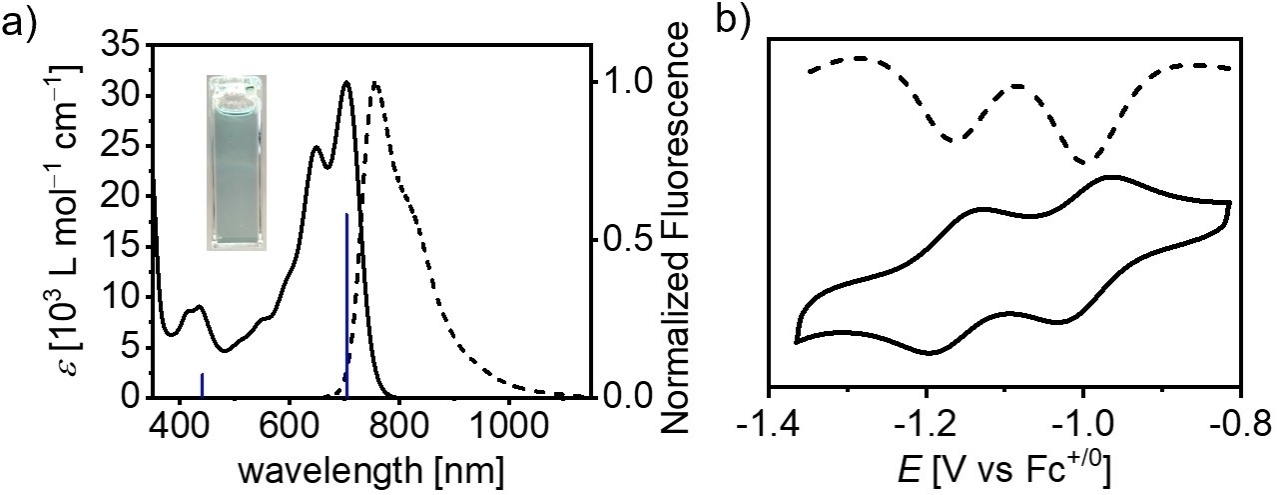
a) UV/Vis absorption (7.49×10^−6^ M, solid line) and emission spectra (*λ*_ex_=645 nm, 1.60×10^−5^ M, dotted line) of **B_2_‐HBP** in *o‐*dichlorobenzene at 298 K. Oscillator strengths obtained by TD‐DFT (B3LYP/6‐311G**) are illustrated with blue bars. Inset: Photographs of solution of **B_2_‐HBP** in ambient light. b) Differential pulse (top) and cyclic voltammogram (bottom) of **B_2_‐HBP** (7×10^−4^ M, 0.1 M (*n*‐Bu)_4_NPF_6_ in *o‐*dichlorobenzene, 298 K).

The electrochemical properties of **2** and **B_2_‐HBP** were studied by cyclic and differential pulse voltammetry in dimethyl sulfoxide (0.1 M (*n*‐Bu)_4_NPF_6_) for **2** or in *o*‐dichlorobenzene (0.1 M (*n*‐Bu)_4_NPF_6_) for **B_2_‐HBP**, based on solubility. Both newly synthesized doubly boron‐doped PAHs show two reversible reductions. Reductions for **2** are observed at potentials −1.34 V and −1.70 V vs. Fc^+/0^, in line with structurally related borinic acid **II**. The fused π‐extended compound **B_2_‐HBP** exhibits significantly anodically shifted (+0.34 V) reductions compared to **2**, at −1.00 V and −1.17 V vs. Fc^+/0^ in *o*‐dichlorobenzene. These are amongst the mildest reduction potentials reported for B‐doped PAHs and are, to our knowledge, the mildest reported for a pristine (unsubstituted) B‐doped PAH.[^2c^, ^2f^, ^3f^, ^3l^] This reflects an unprecedented low LUMO level for **B_2_‐HBP** which validates our DFT‐calculated design. Interestingly **B_2_‐HBP** is easier to reduce than the optimized non‐fused derivative **III** (−1.18 V and −1.48 V vs. Fc^+/0^ in *o*‐dichlorobenzene) from our previous report.^[6b]^ The general trend of the experimentally determined reduction potentials are in good agreement with calculated LUMO levels, with borinic acid **2** (−2.98 eV) exhibiting the highest and **B_2_‐HBP** (−3.46 eV) the lowest LUMO levels, respectively. It is worth noting that **B_2_‐HBP** LUMO levels are effectively lowered with respect to **2** upon ring fusion. This contrasts with previously reported compound **IV** (−1.73 V),^[5c]^ which retains an essentially identical first reduction compared to its non‐ring‐fused derivative (−1.72 V),^[13]^ as well as other molecules that employ methylene tethers[^3e^, ^5e^] to fuse boron‐containing π‐systems.

Crystals of **B_2_‐HBP** suitable for X‐ray crystallography were grown via physical vapour transport (PVT) and studied by synchrotron X‐ray diffraction.^[14]^ Nanographene **B_2_‐HBP** crystallized in the *C*2/*c* space group with an inversion centre in the middle of its central six‐membered ring. The structure is well in agreement with the geometry optimized structure from DFT calculations. Based on further DFT calculations, the *C_i_‐*symmetric and *C_2_*‐symmetric conformations are nearly equal in energy (Δ*G*_diff_=1.55 kJ mol^−1^), with the *C_2_*‐symmetric conformer slightly favoured. The observed *C_i_*‐symmetric conformation is likely the result of crystal packing forces (details in Supporting Information, Figures S29). As anticipated from its broad and moderately planar π‐surface, **B_2_‐HBP** shows 1D columnar π‐stacking with significant overlap of π‐systems over the entire molecule with interplanar distances of 3.4 Å. This crystal packing is similar to that found for **IV**. In contrast, the non‐fused counterpart **III** and B‐doped nanographene **I** do not show any π‐π interaction in the solid state due to sterically demanding mesityl or alkoxy/aryloxy substituents.

Analysis of bond lengths gave insight into the π‐conjugation of **B_2_‐HBP**. In the newly formed six‐membered ring (ring B in Figure 4), the bond lengths of B(1)−C(21) and C(11)−C(12) are shorter than corresponding single bonds in non‐fused analogue **III** (see Supporting Information, Figure S31b),^[6b]^ indicating an extension of π‐conjugation.[^3e^, ^4^, ^5e^, ^5i^, ^5j^, ^15^] Notable changes occurred in ring D, where the bond length of B(1)−C(12) (1.519(9) Å) is significantly shorter than the corresponding B−C bond in **III** (1.545(2) Å) and a single bond of boron and aromatic carbon (B−C_Ar_, 1.556 Å).^[16]^ This is indeed as short as the shortest B−C bond in a neutral aromatic 1,4‐diborabenzene (1.522(3) Å).^[17]^ In contrast, the adjacent bond C(12)−C(13) (1.390(7) Å) is longer than corresponding bond in **III** (1.360(2) Å) and nearly the same as an ideal aromatic carbon−carbon bond (C≃C, 1.384 Å).^[16]^ The B−C and C−C bonds of the central anthracene core (rings E, F and E′) remain unaffected. These observations suggest a higher delocalization of the electrons of C(12)−C(13) bond into the empty p‐orbital of the boron centre. This is also indicated by the larger HOMO orbital coefficients around the boron centre than around the C(12)−C(13) bond for **B_2_‐HBP** compared to non‐fused analogue **III** (Figure 4 and Supporting Information, Figure S25). This allylic electron delocalization (B(1)≃C(12)≃C(13)) of **B_2_‐HBP** can be further substantiated by nucleus‐independent chemical shifts (NICS)^[18]^ and anisotropy of the induced current‐density (AICD)^[19]^ (details in Supporting Information, Figure S30). The central anthracene moiety (rings E, F and E′) of **B_2_‐HBP** exhibits clockwise ring current with large negative NICS(1)_zz_ values between −19.9 and −22.2 ppm, indicating high aromaticity. Similarly, the outer biphenyl moieties (rings A and C) have high aromaticity. On the other hand, the two boracycles (rings B and D) show counterclockwise ring currents with positive NICS(1)_zz_ values of 7.2 ppm for the outer ring B and 5.5 ppm for inner ring D, thereby leaving out the allylic BC_2_ moiety (B(1)≃C(12)≃C(13)) from clockwise ring currents.

**Figure 4:**
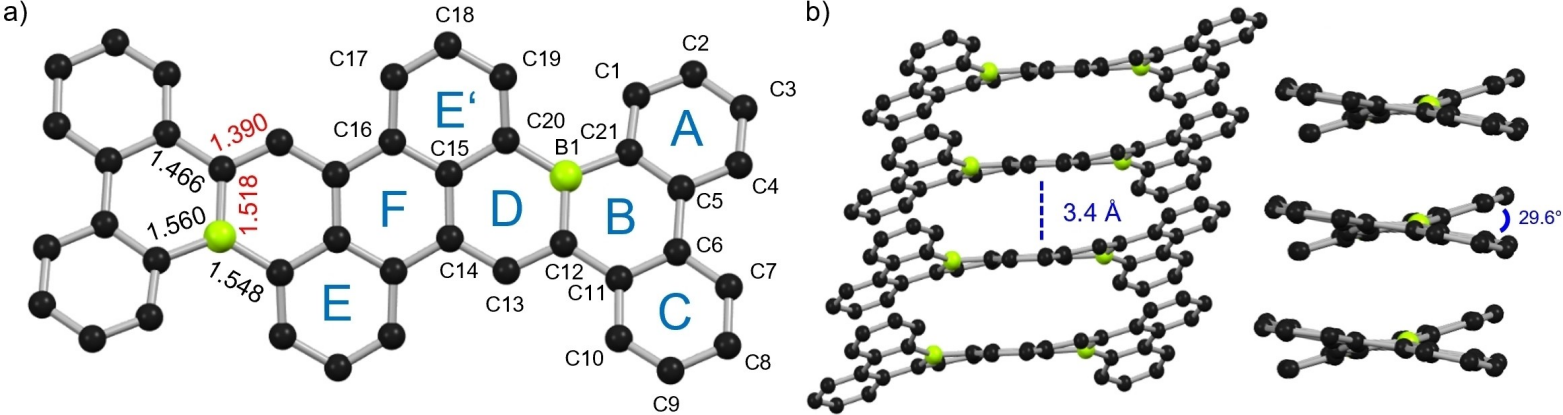
a) Top view of solid‐state molecular structure of **B_2_‐HBP** with selected bond lengths in Å. b) Side view of 1D solid‐state packing of **B_2_‐HBP** with interplanar distances and torsion angle of the [4]helicene unit (C(1)−C(21)−C(20)−C(19)). C atoms: black, B atoms: yellow‐green; H atoms omitted for clarity.

In summary, we have introduced a new fully fused low‐LUMO, pristine B‐doped nanographene. We used geometry‐optimized (TD‐)DFT calculations to predict the optoelectronic properties of **B_2_‐BHP**, which included appealing electron‐accepting ability and molecular geometry. We successfully synthesized fully fused **B_2_‐BHP** from a simple alkene precursor through six net C−H borylations. As predicted by DFT calculations, **B_2_‐BHP** shows remarkably facile reductions and long wavelength absorption in solution as well as emission in the NIR. We could therefore show that simple DFT calculations are an efficient tool to predict the optoelectronic properties of B‐doped nanographenes prior to synthetic efforts. By comparison with non‐fused **III**, we could gain insights into the influence of molecular geometry on frontier molecular orbitals and relate this to observed optoelectronic properties. Investigation by X‐ray crystallography, NICS and AICD calculations revealed infinite 1D columnar packing and an effective π‐conjugation over the whole molecule with allylic B≃C≃C delocalization. These features might contribute to the distinct optoelectronic properties of **B_2_‐BHP** in comparison to other doubly B‐doped nanocarbons. Our validated design‐and‐synthesis approach may therefore provide access to new and promising B‐doped PAHs with tailored properties for electronic or optical applications.

## Conflict of interest

The authors declare no conflict of interest.

## Supporting information

As a service to our authors and readers, this journal provides supporting information supplied by the authors. Such materials are peer reviewed and may be re‐organized for online delivery, but are not copy‐edited or typeset. Technical support issues arising from supporting information (other than missing files) should be addressed to the authors.

Supporting Information

## Data Availability

The data that support the findings of this study are available from the corresponding author upon reasonable request.
