# Supplementary material for: 12b,24b‐Diborahexabenzo[a,c,fg,l,n,qr]pentacene: A Low‐LUMO Boron‐Doped Polycyclic Aromatic Hydrocarbon
Source: Angew Chem Int Ed Engl. 2022 Jan 3;61(8):e202115746. doi: 10.1002/anie.202115746 (PMC9305547; doi:10.1002/anie.202115746)
Supplement: Supplementary file 1 — Supporting Information [file ANIE-61-0-s001.pdf]

Angewandte Chemie
Eine Zeitschrift der Gesellschaft Deutscher Chemiker

Supporting Information

12b,24b-Diborahexabenzo[*a*,*c*,*fg*,*l*,*n*,*qr*]pentacene: A Low-LUMO Boron-Doped Polycyclic Aromatic Hydrocarbon

*C. Mützel, J. M. Farrell, K. Shoyama, F. Würthner**

Table of Contents

1) Materials and Methods 2
2) Synthetic Procedures 3
3) NMR Spectra 8
4) Mass spectra 14
5) Absorption and emission spectra 15
6) Differential pulse and cyclic voltammetry 16
7) Computations 18
8) X-ray crystallography 32
9) References 33

1) Materials and Methods

General remarks: Where indicated, synthetic manipulations were carried out under an atmosphere of dry, O_2-free N_2 in a glovebox (M. Braun Inertgas-Systeme GmbH) using oven-dried glassware. 1,3-diisopropylimidazol-2-ylidene borane,[1] 2-biphenylcarbaldehyde[2] and 9,10-bis(diethylphosphonomethyl)anthracene[3] were prepared according to literature procedures. Aluminium (III) chloride, 2,6-dichloropyridine, bis(trifluoromethylsulfonyl)imide and 2,2,6,6-tetramethylpiperidine-1-oxyl free radical (TEMPO radical) were obtained from TCI and used without further purification. Anhydrous chlorobenzene (CB) and 1,2-dichlorobenzene (*o*-DCB) were obtained from Sigma Aldrich and dried over 4 Å molecular sieves before use. DMSO was dried over 4 Å molecular sieves before use. THF and dichloromethane were purified with a Grubbs-type column system manufactured by Innovative Technology. Deuterated solvents were obtained from commercial sources and used without further purification. Anhydrous hexane was obtained from Sigma Aldrich and used without further purification. All other solvents for spectroscopic measurements were spectroscopic grade and used without further purification. Column chromatography was performed with commercial glass columns using silica gel 60M (particle size 0.04–0.063 mm). All other reagents and solvents were obtained from commercial sources and used without further purification.

UV/Vis absorption spectra were recorded on a Jasco V-670 or Jasco V-770 spectrophotometer for solution phase measurements.

Fluorescence spectra were recorded on an Edinburgh Instruments FLS980 fluorescence spectrometer. Relative fluorescence quantum yields were determined using the comparative method at four excitation wavelengths with respect to standards: rhodamine 101 in EtOH and 1,1',3,3,3',3'-hexamethylindotricarbocyanine iodide in EtOH.[4] Time-resolved measurements were performed with Edinburgh S3 Instruments picosecond pulsed laser diodes and a TCSPC detection unit.

NMR spectra were recorded on Bruker Avance III HD 400 or Bruker Avance III HD 600 spectrometers. Chemical shifts are listed in parts per million and are given relative to $SiMe_4$ and referenced to a residual solvent signal (^1H, ^{13}C). Coupling constants (*J*) are quoted in Hertz (Hz). ^{11}B signals for boron-containing compounds could not be observed due to broadening and/or poor solubility.

High resolution mass spectrometry was carried out on a Bruker Daltonics micrOTOF focus or on a Bruker Daltonics ultrafleXtreme instrument.

Cyclic voltammetry was carried out using a standard commercial electrochemical analyzer (EC epsilon; BAS Instruments, UK) with a three-electrode single-compartment cell. The supporting electrolyte tetrabutylammonium hexafluorophosphate ($(n\text{-Bu})_4NPF_6$) was prepared according to the literature,[5] and recrystallized from ethanol/water. The measurements were carried out using ferrocene (Fc) as an internal standard for the calibration of the potential. Potentials of irreversible redox events were determined by square

wave voltammetry experiments. An Ag/AgCl reference electrode was used. A Pt disc and a Pt wire were used as working and auxiliary electrodes, respectively.

Single crystal X-ray diffraction data were collected at the P11 beamline at DESY. The diffraction data were collected by a single 360° scan ϕ sweep at 100 K. The diffraction data were indexed, integrated, and scaled using the XDS program package.[6] The structure were solved using SHELXT,[7] expanded with Fourier techniques and refined using the SHELX software package.[8] Hydrogen atoms were assigned at idealized positions and were included in the calculation of structure factors. All non-hydrogen atoms were refined anisotropically. Crystallographic data have been deposited with the Cambridge Crystallographic Data Centre as supplementary publication no. CCDC 2122085 (**B$_2$-HBP**). These data can be obtained free of charge from The Cambridge Crystallographic Data Centre via www.ccdc.ac.uk/data.request/cif.

Computational details. Geometry optimizations were performed by the density functional theory (DFT) calculations employing B3LYP as functional[9] and 6-311G** as basis set[10] as implemented in the Gaussian 09 program package.[11] The optimized geometries were confirmed to have no negative frequency by frequency calculations. Time-dependent density functional theory (TD-DFT) calculations were performed on the geometry-optimized structures employing the same basis set and functional as for the geometry optimizations. The absorption spectra were simulated by the GaussView 5 visualization software package.[12] NICS(1)$_{zz}$ values were calculated using the NMR chemical shift simulated by the GIAO method. The visualization of AICD was performed by the program package provided by Prof. R. Herges.

2) Synthetic Procedures

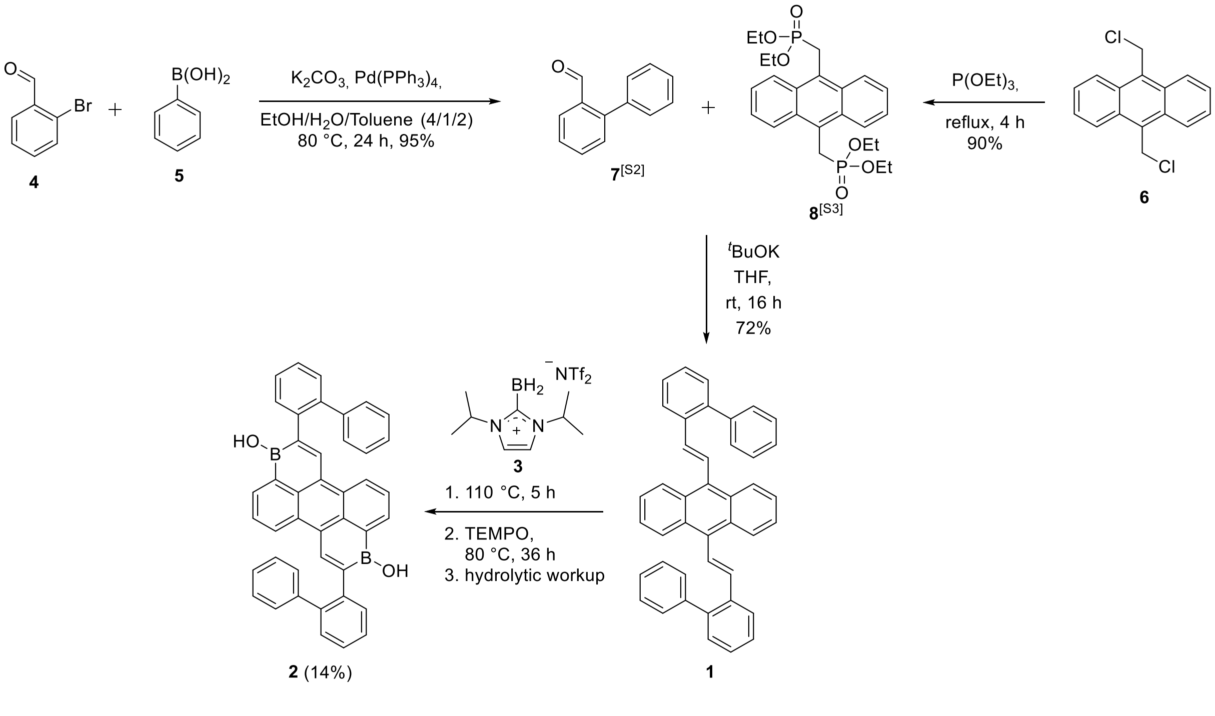

Scheme S1. Reaction scheme for the synthesis of precursor **2**.

9,10-Bis[(*E*)-2-(biphenyl-2-yl)vinyl]anthracene (1)

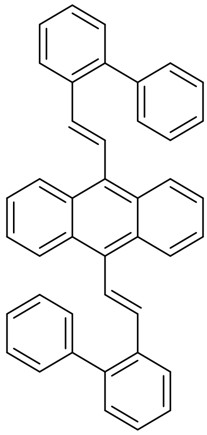

1

9,10-Bis(diethylphosphonomethyl)anthracene (1.00 g, 2.09 mmol) and potassium *tert*-butoxide (1.88 g, 16.7 mmol) were dissolved in dry THF (45 mL). Then 2-biphenylcarbaldehyde (952 mg, 5.23 mmol) was added and the solution was stirred at room temperature for 16 h. The resulting precipitate was filtered, washed with methanol and recrystallized from chloroform to yield **1** as a yellow solid (890 mg, 80%, 1.66 mmol).

^{1}H NMR (400.1 MHz, CD_2Cl_2, 298 K): δ 8.31 (dd, 2H, $^3J_{HH}$ = 6.8 Hz, $^3J_{HH}$ = 6.8 Hz, Ar-*H*), 8.08 (d, 2H, $^3J_{HH}$ = 6.5 Hz, Ar-*H*), 7.85 (d, 2H, $^3J_{HH}$ = 16.3 Hz, Ar-*H*), 7.52 (td, 2H, $^3J_{HH}$ = 7.68 Hz, $^4J_{HH}$ = 1.64 Hz, Ar-*H*), 7.46–7.38 (m, 12H, Ar-*H*), 7.34 (t, 2H, $^3J_{HH}$ = 7.2 Hz, *Ar*-H), 7.26 (t, 2H, $^3J_{HH}$ = 7.36 Hz, Ar-*H*), 6.83 (d, 2H, $^3J_{HH}$ = 16.4 Hz, Ar-*H*). **^{13}C NMR** (100.6 MHz, CD_2Cl_2, 298 K): δ 141.7, 141.2, 136.8, 136.0, 133.1, 130.7, 130.1, 129.7, 128.4, 128.2, 128.1, 127.4, 126.7, 126.6, 126.5, 125.5. **M.P.:** 270 °C. **HRMS** (ESI-TOF, positive mode): m/z: 534.2352, $[M]^+$ calculated for $C_{42}H_{30}$: 534.23420.

2,8-Bis(biphenyl-2-yl)-3,9-diboraperylene-3,9-diol (2)

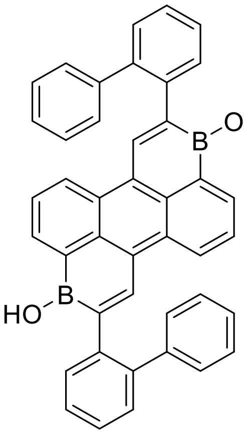

2

In a nitrogen-filled glovebox 1,3-diisopropylimidazol-2-ylidene borane (299 mg, 1.80 mmol) and bis(trifluoromethylsulfonyl)imide (515 mg, 1.83 mmol) were dissolved in chlorobenzene (10 mL) and the mixture was stirred at room temperature for 90 min. 9,10-Bis[(*E*)-2-(biphenyl-2-yl)vinyl]anthracene (401 mg, 750 µmol) was added and the flask was sealed with a dropping funnel containing TEMPO (498 mg, 4.60 mmol) dissolved in 10 mL chlorobenzene. The apparatus was brought out of the glovebox and heated at 110 °C for 6 h. After cooling to 80 °C, the TEMPO solution was added and the mixture was stirred at 80 °C for another 38 h. The reaction was cooled to room temperature and the solvent was removed under reduced pressure. Purification with column chromatography (eluent: toluene/dichloromethane 3/2) yielded **2** as a black solid (61.7 mg, 105 µmol, 14%).

^{1}H NMR (400.1 MHz, DMSO-d_6, 298 K): δ 9.21 (s, 2H, B-O*H*), 8.85 (d, 2H, $^{3}J_{HH}$ = 8.68 Hz, Ar-*H*), 8.70 (s, 2H, Ar-*H*), 8.47 (d, 2H, $^{3}J_{HH}$ = 6.64 Hz, Ar-*H*), 7.75 (dd, 2H, $^{3}J_{HH}$ = 8.72 Hz, $^{3}J_{HH}$ = 6.64 Hz, Ar-*H*), 7.58 (dd, 2H, $^{3}J_{HH}$ = 6.68 Hz, $^{4}J_{HH}$ = 1.88 Hz, Ar-*H*), 7.47–7.43 (m, 6 H, Ar-*H*), 7.40–7.37 (bdd, 4 H, $^{3}J_{HH}$ = 8.48 Hz, $^{4}J_{HH}$ = 1.48 Hz, Ar-*H*), 7.18 (btd, 4 H, $^{3}J_{HH}$ = 7.60 Hz, $^{4}J_{HH}$ = 1.56 Hz, Ar-*H*), 7.10 (td, 2 H, $^{3}J_{HH}$ = 7.32 Hz, $^{4}J_{HH}$ = 1.36 Hz, Ar-*H*). **^{13}C NMR** (100.1 MHz, DMSO-d_6, 298 K): δ 147.0, 142.4, 142.23, 142.18, 140.3, 136.32, 136.26, 132.4, 129.9, 129.8, 129.6, 129.4, 128.6, 128.5, 127.8, 127.3, 126.9, 126.6, 126.4. **^{11}B NMR** (128 MHz, DMSO-d_6, 298 K): not observed. **M.P.:** 260 °C. **HRMS** (MALDI-TOF, positive mode): m/z: 586.22895, $[M]^+$ calculated for $C_{42}H_{32}B_2O_2$: 586.22699. **CV** (7×10^{-4} M, 0.1 M (n-Bu)$_4$NPF$_6$ in DMSO, vs. $Fc^{+/0}$, 298 K): $E_{1/2\ red}$ (**2**/**2**$^{-}$) = −1.36 V, $E_{1/2\ red}$ (**2**$^{-}$/**2**$^{2-}$) = −1.70 V. **UV/Vis** (7.57×10^{-6} M in dichloromethane, 298 K): λ_{max} (ε_{max}) = 560 nm (26600), 523 nm (20700), 407 nm (4700), 402 nm (4800), 289 nm (40100). **Fluorescence** (1.85×10^{-6} M in dichloromethane, 298 K): λ_{max} = 601 nm (Φ = 0.70).

12b,24b-Diborahexabenzo[*a*,*c*,*fg*,*l*,*n*,*qr*]pentacene (B_2-HBP)

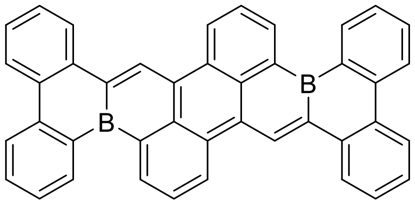

B_2-HBP

BBr_3 (14.2 µL, 149 µmol, 5 equiv.) was slowly added to borinic acid **2** (17.5 mg, 29.9 µmol) in dry dichloromethane (4 mL). The mixture changed the colour from red to blue. The solution was stirred at room temperature for 30 min. The solvent and excess of BBr_3 were removed under vacuum and the residue was suspended in dry dichloromethane (3 mL). 2,6-Dichloropyridine (9.06 mg, 61.2 µmol, 2.05 equiv.) and $AlCl_3$ (8.36 mg, 62.7 µmol, 2.1 equiv.) were added and the suspension was stirred at room temperature for 1 h. The colour of reaction mixture changed from dark blue to light blue. The solvent was removed and the residue was washed with hexane, toluene, dichloromethane. The crude product was purified by sublimation (370 °C, 10^{-4} mbar) to yield **B_2-HBP** as a black solid (1.00 mg, 1.82 µmol, 6%).

^{1}H NMR (400.1 MHz, $C_6D_4Cl_2$, 298 K): δ 9.59 (s, 2H, Ar-*H*), 9.21 (d, 2H, $^3J_{HH}$ = 8.44 Hz, Ar-*H*), 8.97 (d, 2H, $^3J_{HH}$ = 6.44 Hz, Ar-*H*), 8.38 (d, 2H, $^3J_{HH}$ = 6.24 Hz, Ar-*H*), 8.29 (d, 2H, $^3J_{HH}$ = 8.68 Hz, Ar-*H*), 8.15–8.12 (m, 4H, Ar-*H*), 7.85 (dd, 2H, $^3J_{HH}$ = 8.68 Hz, $^3J_{HH}$ = 6.68 Hz, Ar-*H*), 7.50 (m, 2H, Ar-*H*), 7.37–7.31 (m, 6H, Ar-*H*). **^{13}C NMR** (100.1 MHz, $C_6D_4Cl_2$, 298 K): Due to poor solubility ^{13}C NMR signals could not be observed. **^{11}B NMR** (128 MHz, $C_6D_4Cl_2$, 298 K): not observed. **HRMS** (MALDI-TOF, negative mode): m/z: 550.20649, $[M]^-$ calculated for $C_{48}H_{40}B_2$: 550.20696. **M.P.:** >350 °C. **CV** (7 × 10^{-4} M, 0.1 M (n-Bu)$_4$NPF$_6$ in *o*-DCB, vs. $Fc^{+/0}$, 298 K): $E_{1/2\ red}$ (**2**/**2**$^-$) = −1.00 V, $E_{1/2\ red}$ (**2**$^-$/**2**$^{2-}$) = −1.17 V. **UV/Vis** (7.49 × 10^{-6} M in *o*-dichlorobenzene, 298 K): λ_{max} (ε_{max}) = 704 nm (31300), 649 nm (24900), 436 nm (9100), 417 nm (8700), 304 nm (35500). **Fluorescence** (1.60 × 10^{-5} M in *o*-dichlorobenzene, 298 K): λ_{max} = 757 nm (Φ = 0.06).

Table S1. Tested reaction conditions for C−H borylations.

Temp [°C]	Solvent	Base	Lewis acid	Oberservations (Yield)
100	Toluene	-	-	No conversion[a]
150	*o*-Dichlorobenzene	-	-	Decomposition
125	Chlorobenzene	-	-	No conversion[a]
125	Chlorobenzene	-	$AlCl_3$	No conversion[a]
100	Toluene	Hünig's base	-	Decomposition
100	Toluene	LiHMDS	-	Decomposition
100	Toluene	2,6-Dichloropyridine	-	No conversion[a]
150	*o*-Dichlorobenzene	2,6-Dichloropyridine	$AlCl_3$	Decomposition
r.t.	Dichloromethane	2,6-Dichloropyridine	$AlCl_3$	Conversion (6%)
r.t.	Dichloromethane	2,6-Di-*t*-butylpyridine	$AlCl_3$	Conversion (3%)
r.t.	Dichloromethane	2,6-Di-*t*-butylpyridine/ 2,6-Dichloropyridine	$AlCl_3$	Conversion (1%)
r.t.	Dichloromethane	2,6-Dichloropyridine	$FeCl_3$	Decomposition
r.t.	Dichloromethane	2,6-Dichloropyridine	$Sc(OTf)_3$	Decomposition

[a] only starting material **2** observed.

3) NMR Spectra

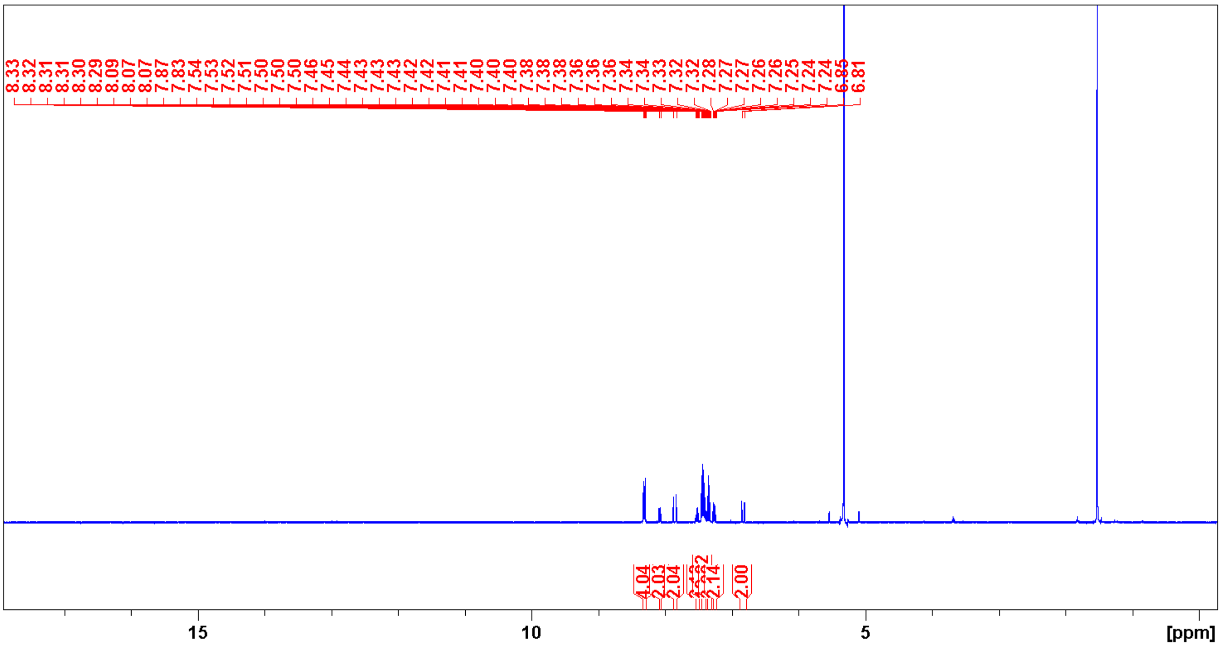

Figure S1. ^{1}H NMR spectrum of **1** (400 MHz, CD_2Cl_2, 298 K).

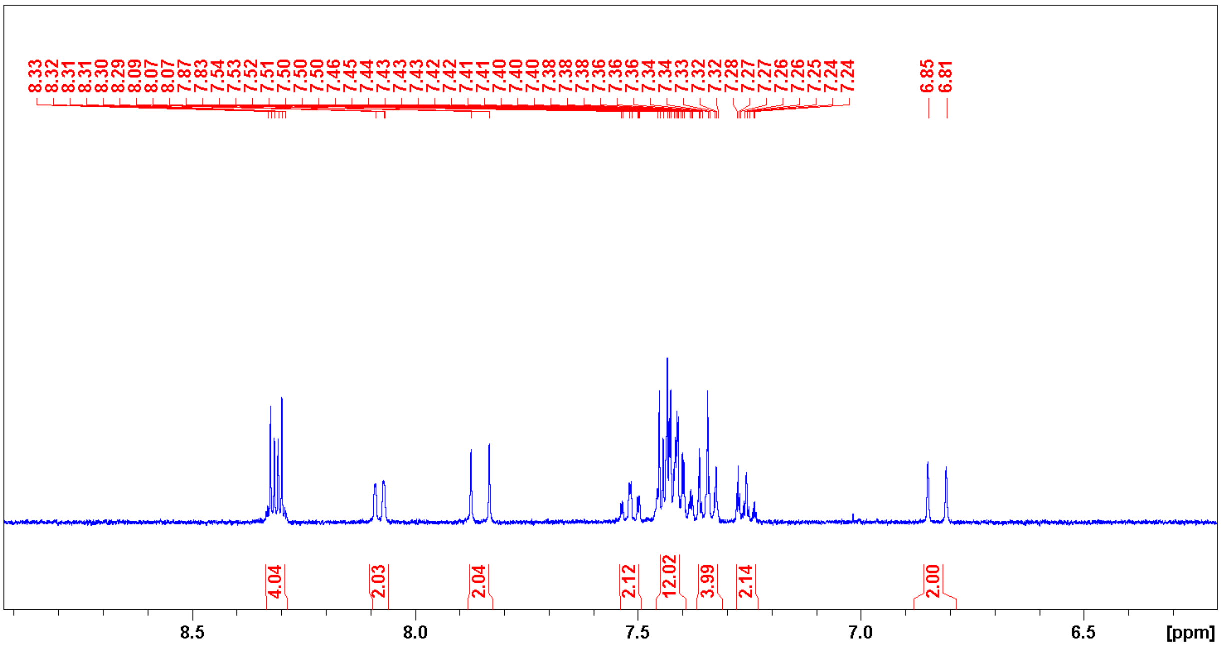

Figure S2. Magnified aromatic region of the ^{1}H NMR spectrum of **1** (400 MHz, CD_2Cl_2, 298 K).

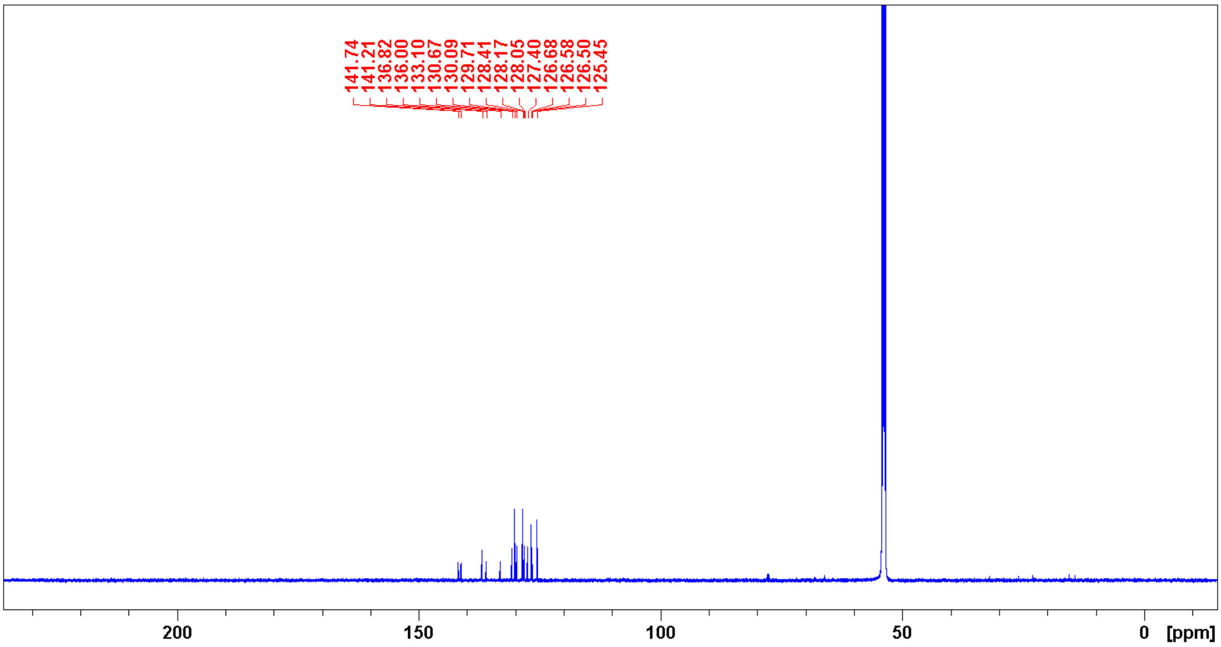

Figure S3. ^{13}C NMR spectrum of **1** (400 MHz, CD_2Cl_2, 298 K).

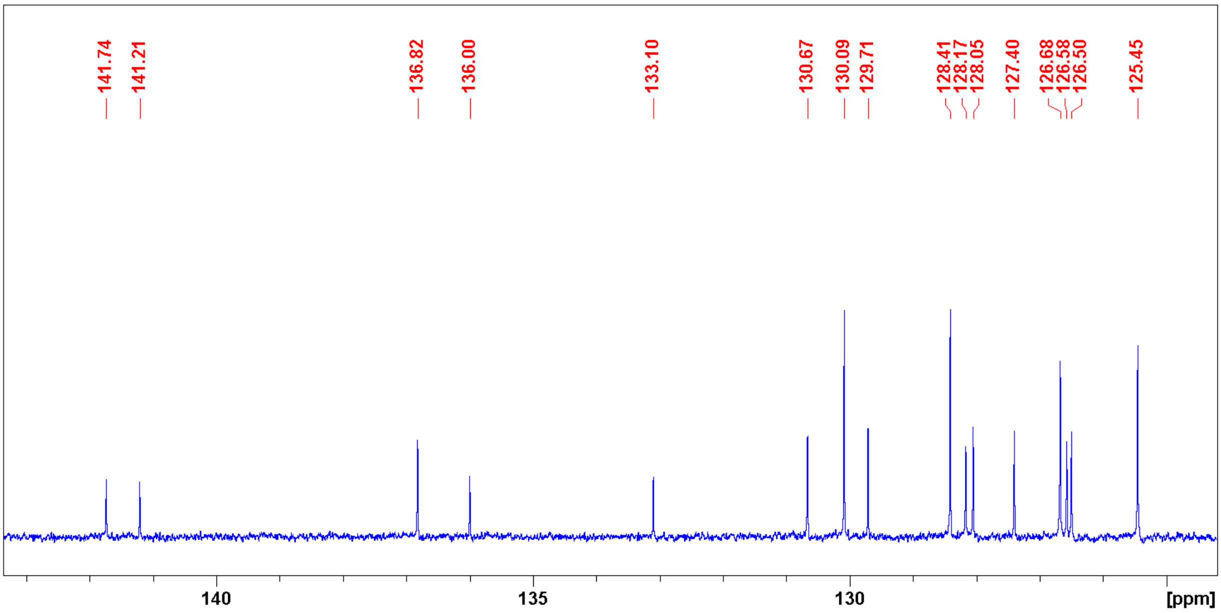

Figure S4. Magnified aromatic region of the ^{13}C NMR spectrum of **1** (400 MHz, CD_2Cl_2, 298 K).

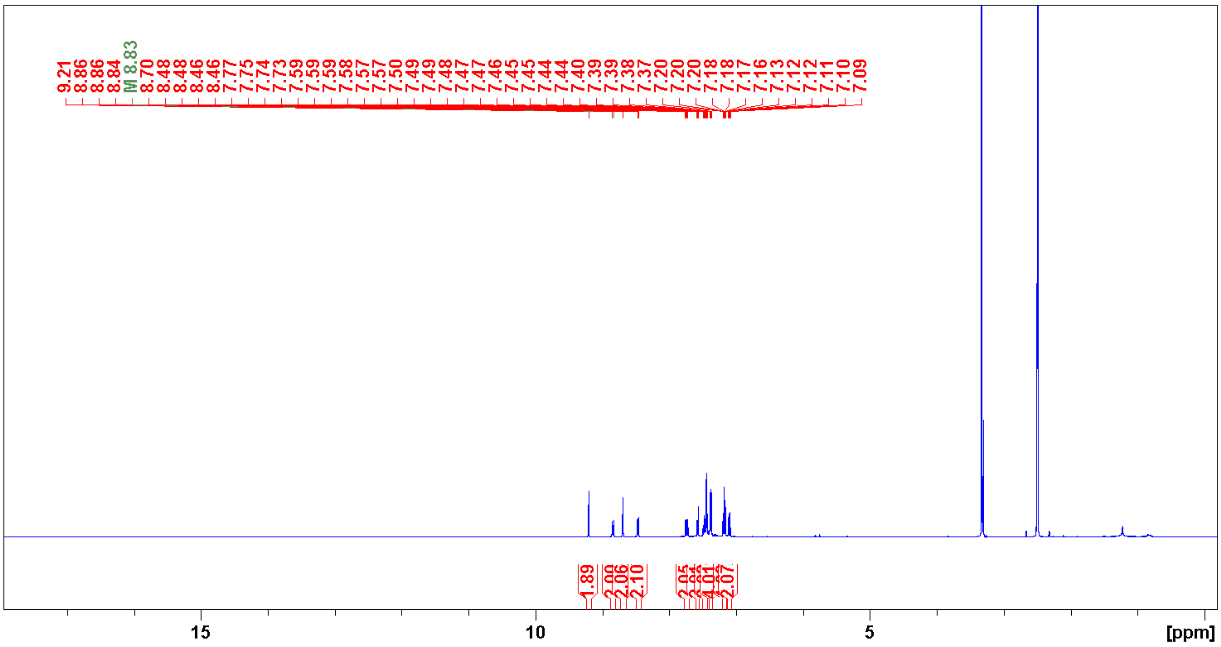

Figure S5. ^{1}H NMR spectrum of **2** (400 MHz, DMSO-d_6, 298 K).

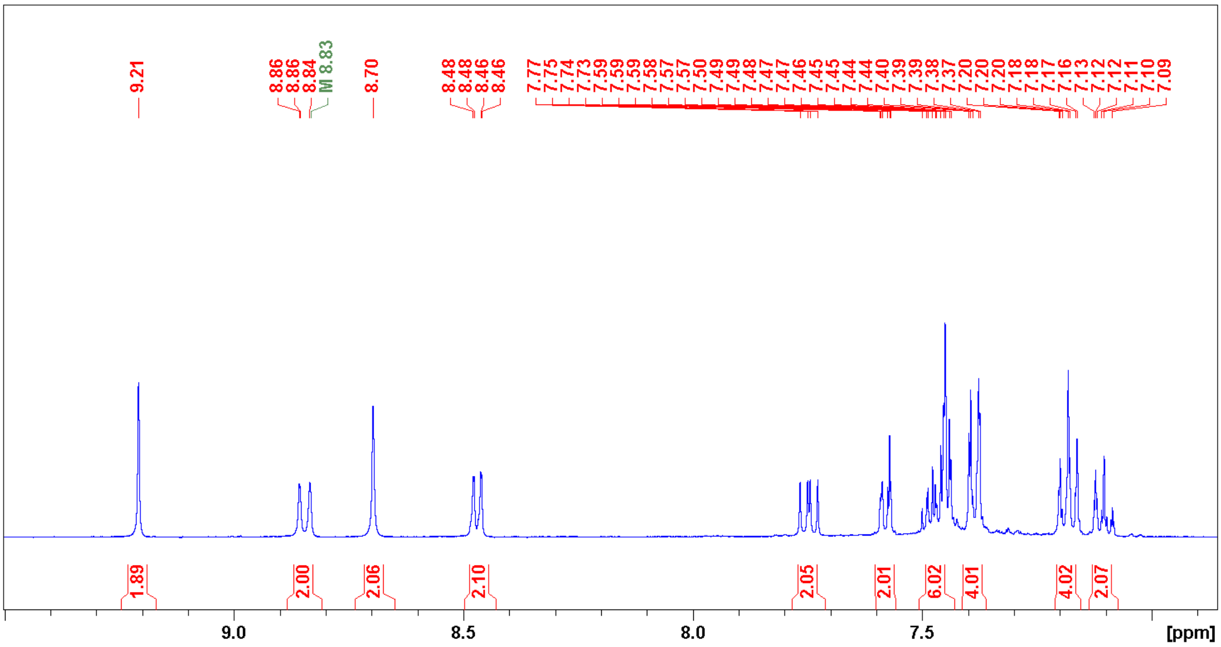

Figure S6. Magnified aromatic region of the ^{1}H NMR spectrum of **2** (400 MHz, DMSO-d_6, 298 K).

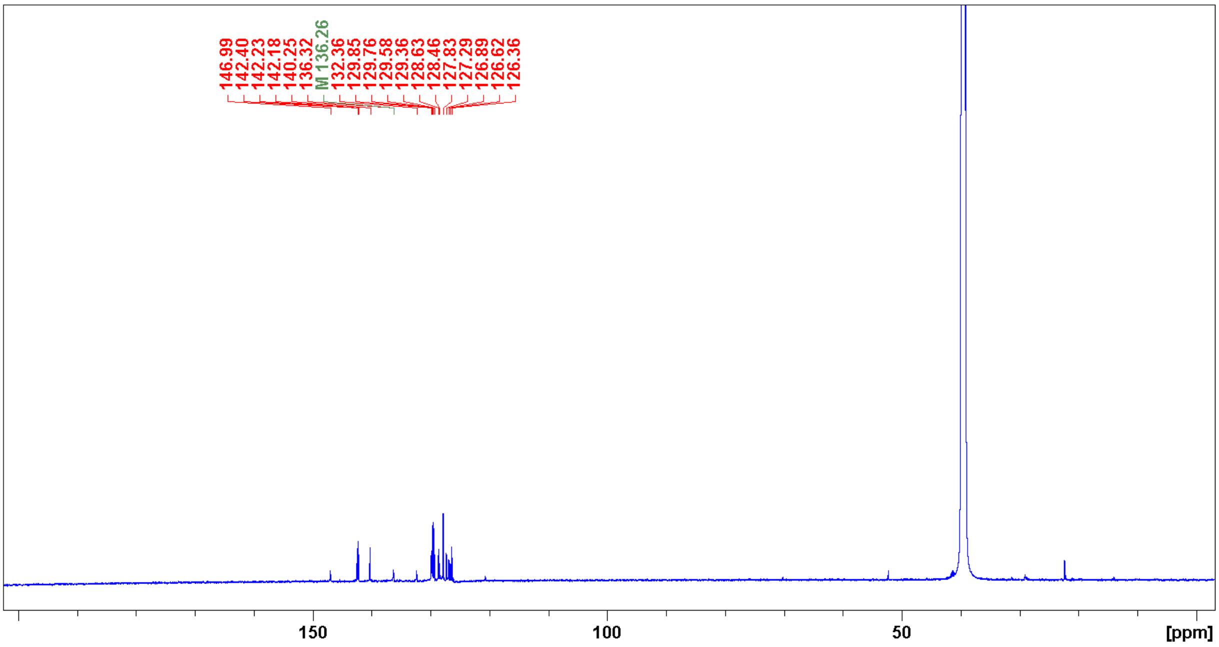

Figure S7. ^{13}C NMR spectrum of **2** (400 MHz, DMSO-d_6, 298 K).

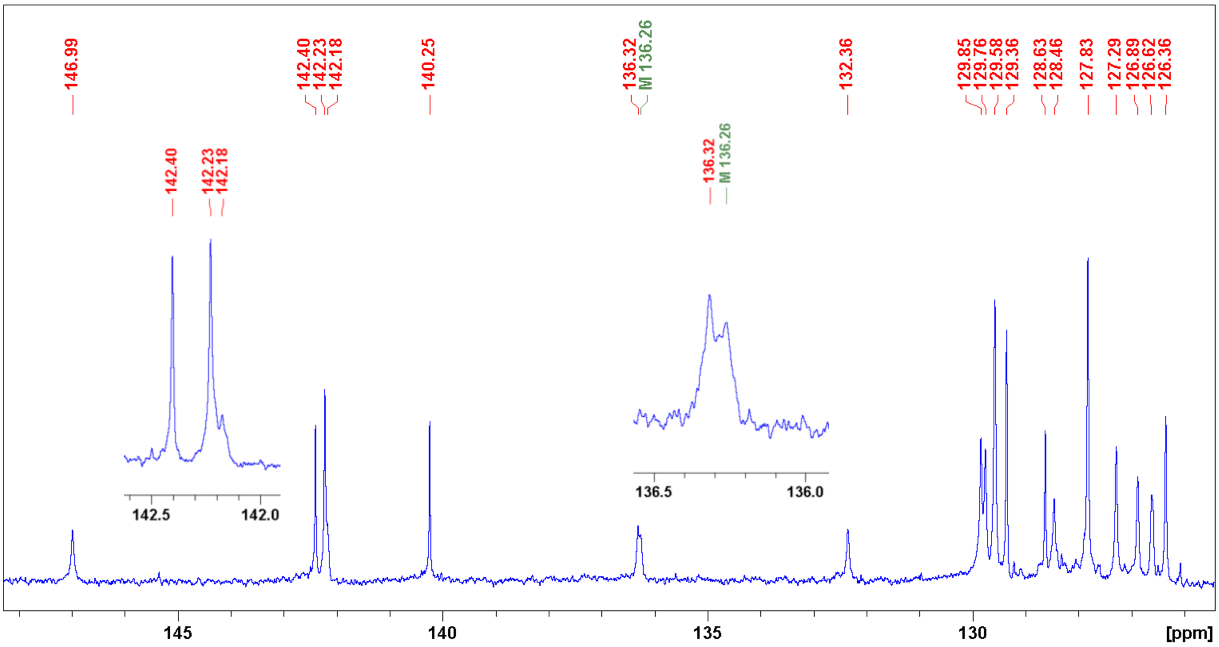

Figure S8. Magnified aromatic region of the ^{13}C NMR spectrum of **2** (400 MHz, DMSO-d_6, 298 K).

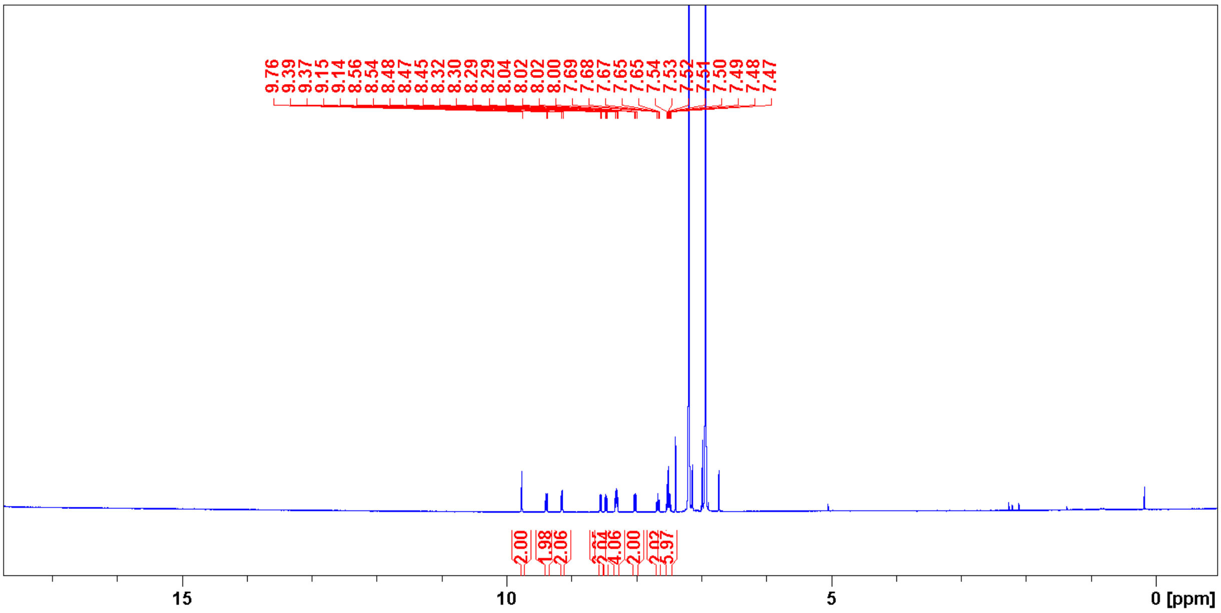

Figure S9. ^{1}H NMR spectrum of **B_2-HBP** (400 MHz, $C_6D_4Cl_2$, 298 K). The peaks between 2 and 3 ppm correspond to impurities in $C_6D_4Cl_2$, which could be confirmed by measuring the solvent only (see Figure S12).

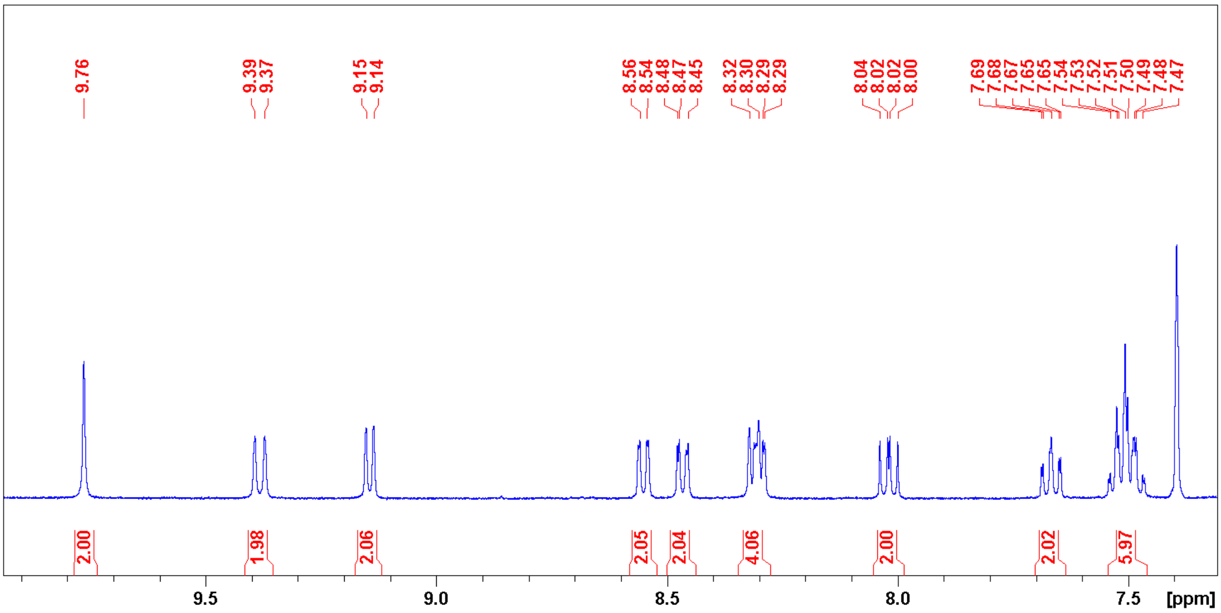

Figure S10. Magnified aromatic region of the ^{1}H NMR spectrum of **B_2-HBP** (400 MHz, $C_6D_4Cl_2$, 298 K).

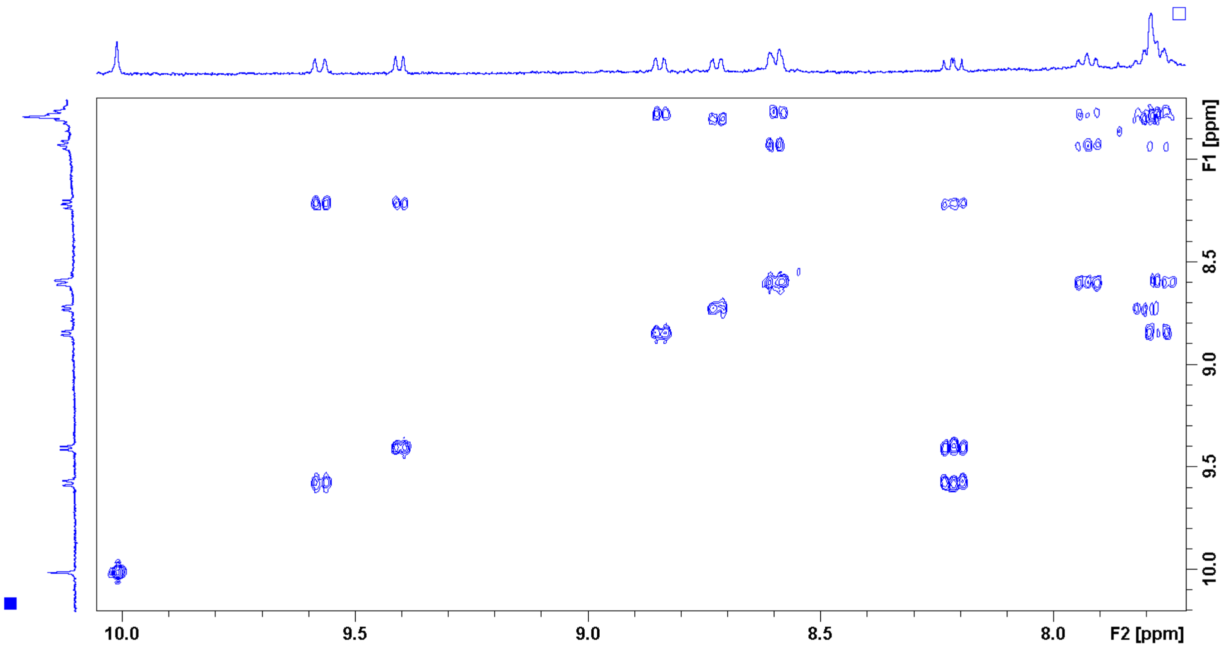

Figure S11. $^1H-^1H$ COSY NMR spectra of **B_2-HBP** (400 MHz, C_6D_5Br, 298 K) showing the aromatic region.

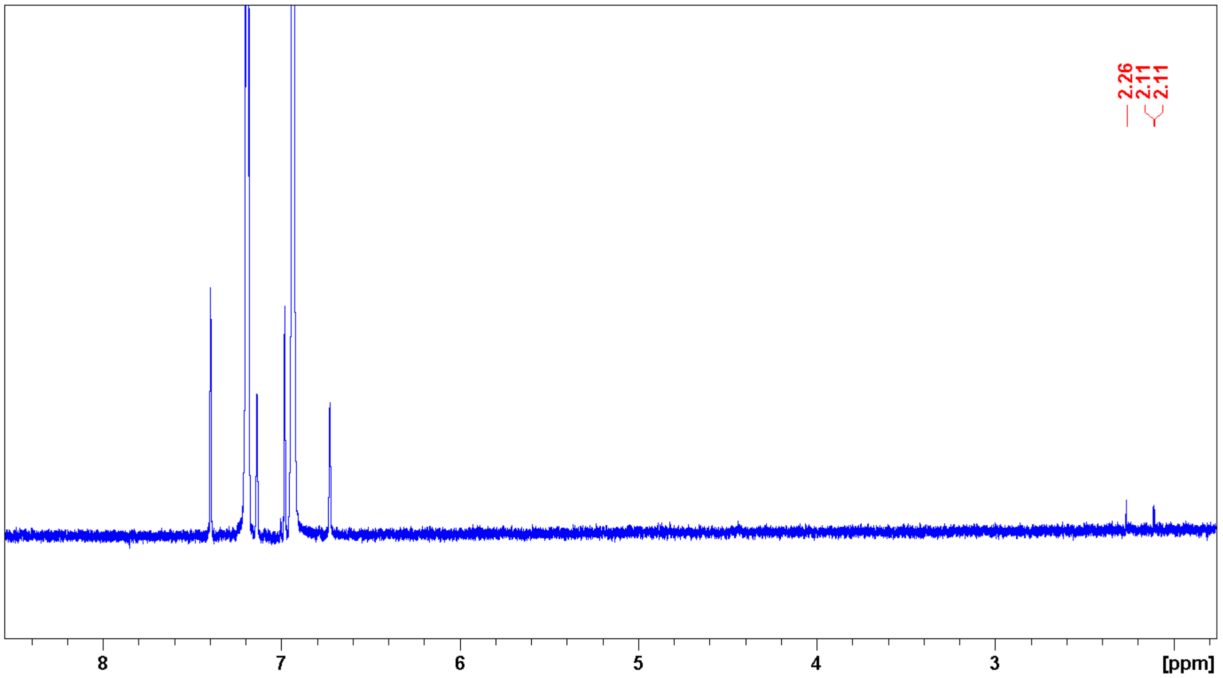

Figure S12. ^1H NMR spectrum of pure deuterated *o*-DCB (400 MHz, $C_6D_4Cl_2$, 298 K) showing the impurities at 2.26 and 2.11 ppm.

4) Mass spectra

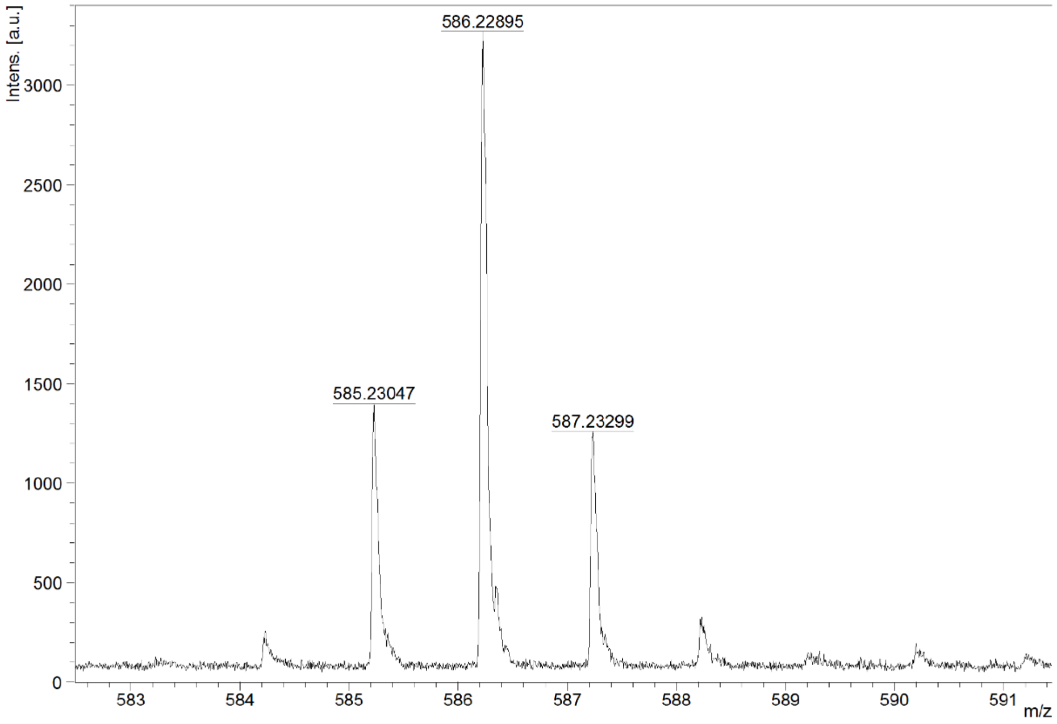

Figure S13. High-resolution mass spectrum of **2** (MALDI-TOF, positive mode).

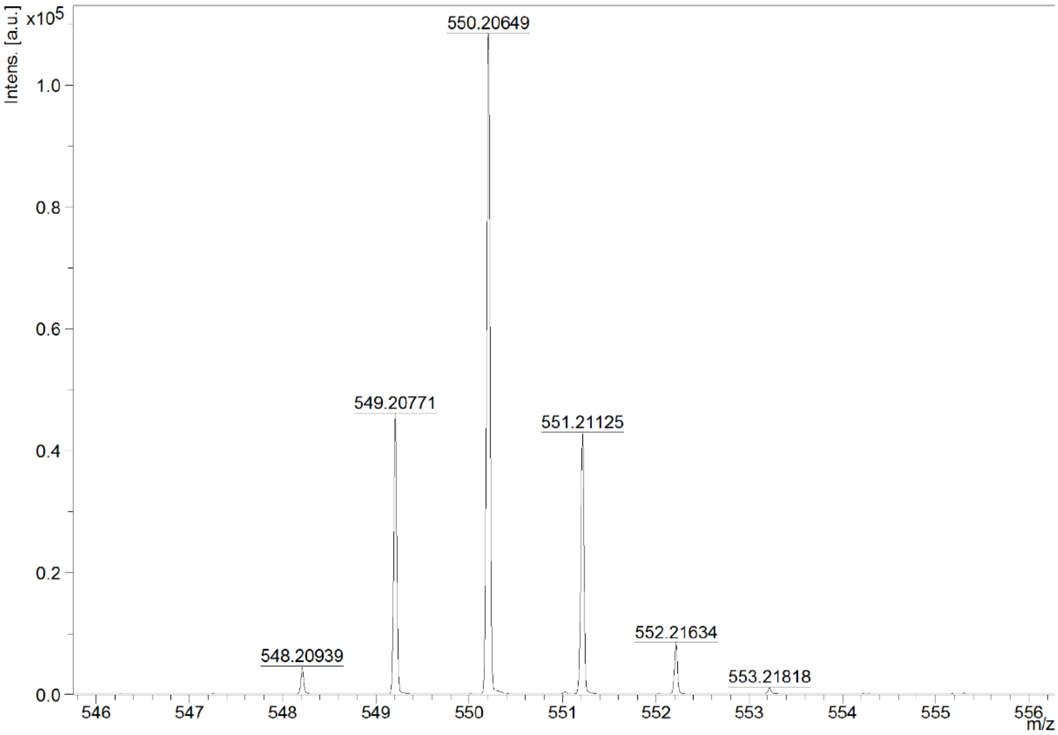

Figure S14. High-resolution mass spectrum of **B_2-HBP** (MALDI-TOF, negative mode).

5) Absorption and emission spectra

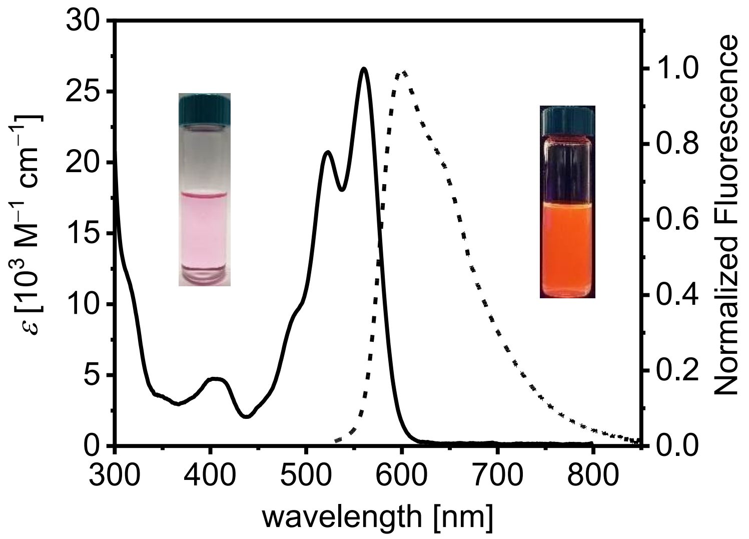

Figure S15. UV/Vis-absorption (7.57×10^{-6} M, solid line) and emission spectra (λ_{ex} = 520 nm, 1.54×10^{-6} M, dotted line) of **2** in CH_2Cl_2 at 298 K. Inset: Photographs of solution of **2** in ambient light (left) and under UV illumination (366 nm).

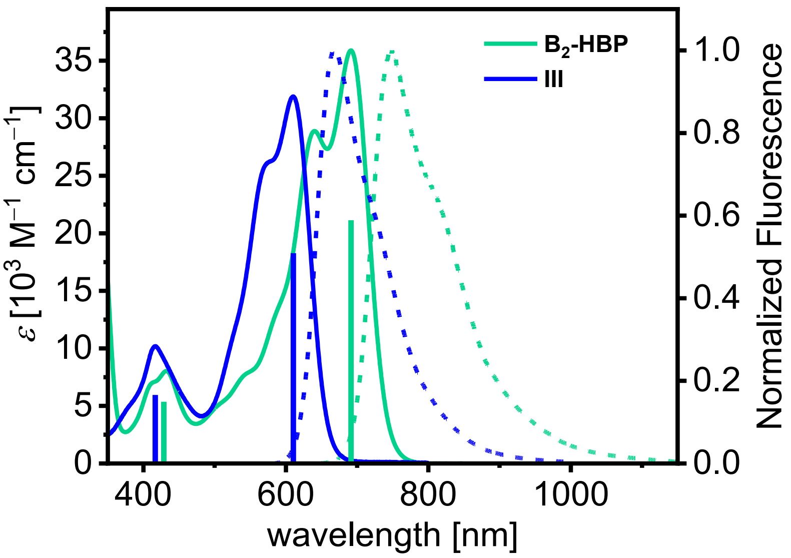

Figure S16. UV/Vis-absorption (~1×10^{-5} M, solid lines) and emission spectra (~1×10^{-6} M, dashed lines) of **III** (dark blue) and **B_2-HBP** (green) in CH_2Cl_2 at room temperature with corresponding transitions and oscillator strengths obtained from TD-DFT calculations (B3LYP/6-311G**).

6) Differential pulse and cyclic voltammetry

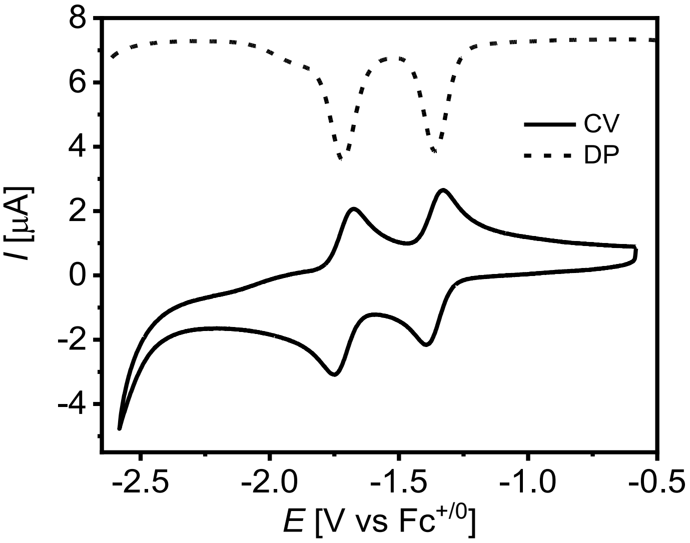

Figure S17. Cyclic and differential pulse voltammograms of **2** (7×10^{-4} M, 0.1 M (n-Bu)$_4$NPF$_6$ in DMSO, 298 K).

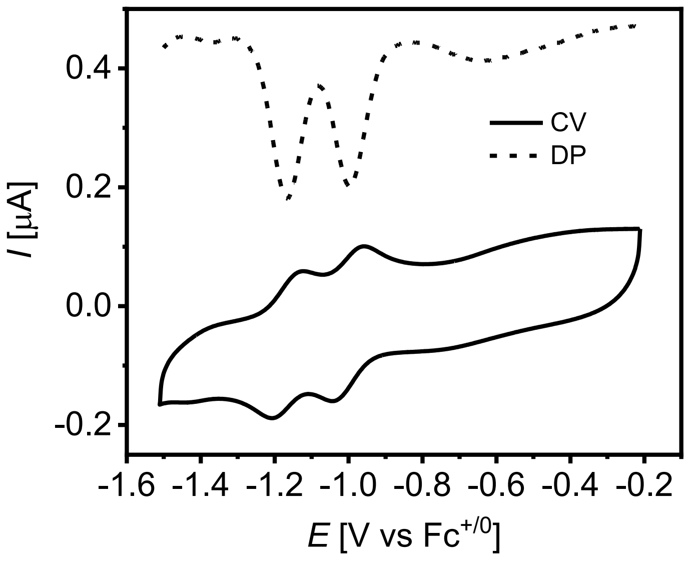

Figure S18. Cyclic and differential pulse voltammograms of **B$_2$-HBP** (7×10^{-4} M, 0.1 M (n-Bu)$_4$NPF$_6$ in o-DCB, 298 K).

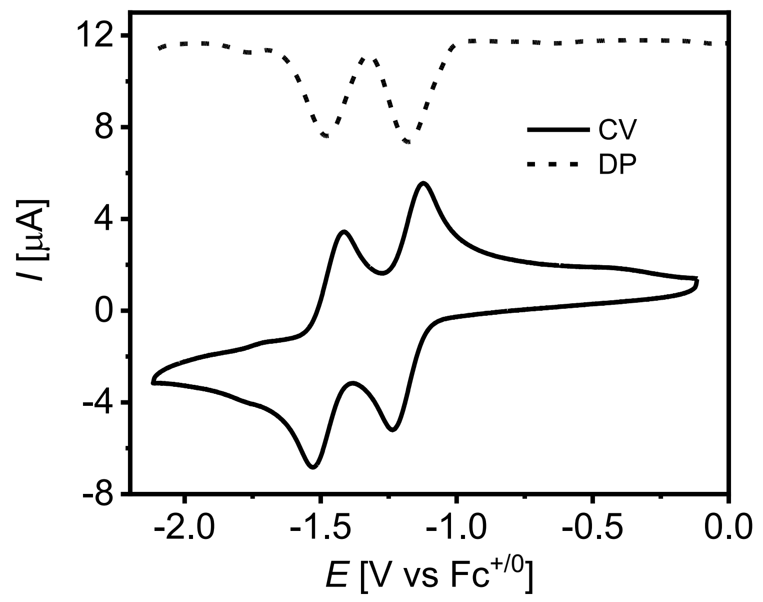

Figure S19. Cyclic and differential pulse voltammograms of **III** (7 × 10^{-4} M, 0.1 M (n-Bu)$_4$NPF$_6$ in o-DCB, 298 K).

Table S2. Summary of optoelectronic properties of **2**, **B$_2$-HBP** and **III**.

compound	λ_{abs} [nm]	ε [M^{-1} cm^{-1}]	λ_{em} [nm]	Stokes Shift [cm^{-1}]	Φ [%]	τ_1 [ns]	First $E_{1/2\ red}$ [V]	Second $E_{1/2\ red}$ [V]
**2**	560[a]	26600[a]	601[a]	1220[a]	70[a]	5.51[a]	−1.34[c]	−1.70[c]
**B$_2$-HBP**	704[b]	31300[b]	757[b]	1000[b]	6[b]	1.00[b]	−1.00[d]	−1.17[d]
	690[a]	35900[a]	745[a]	1100[a]	–	–	–	–
**III**	611[a]	31900[a]	668[a]	1400[a]	74[a]	5.60[a]	−1.18[d]	−1.48[d]

[a] Optical measurements carried out at 298 K in CH_2Cl_2. [b] Optical measurements carried out at 298 K in o-dichloro-benzene. [c] Electrochemical measurements carried out at 298 K in 0.1 M (n-Bu)$_4$NPF$_6$ in DMSO. [d] Electrochemical measurements carried out at 298 K in (n-Bu)$_4$NPF$_6$ 0.1 M o-dichlorobenzene.

7) Computations

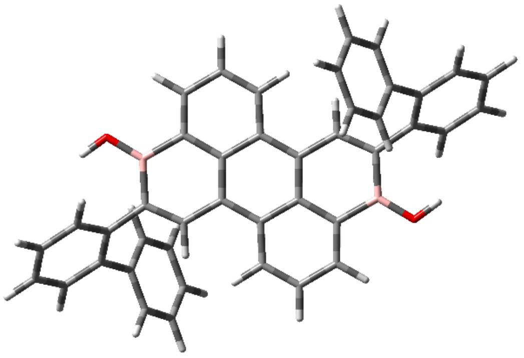

Figure S20. Geometry-optimized structure of **2** by DFT calculations at the B3LYP/6-311G** level of theory.

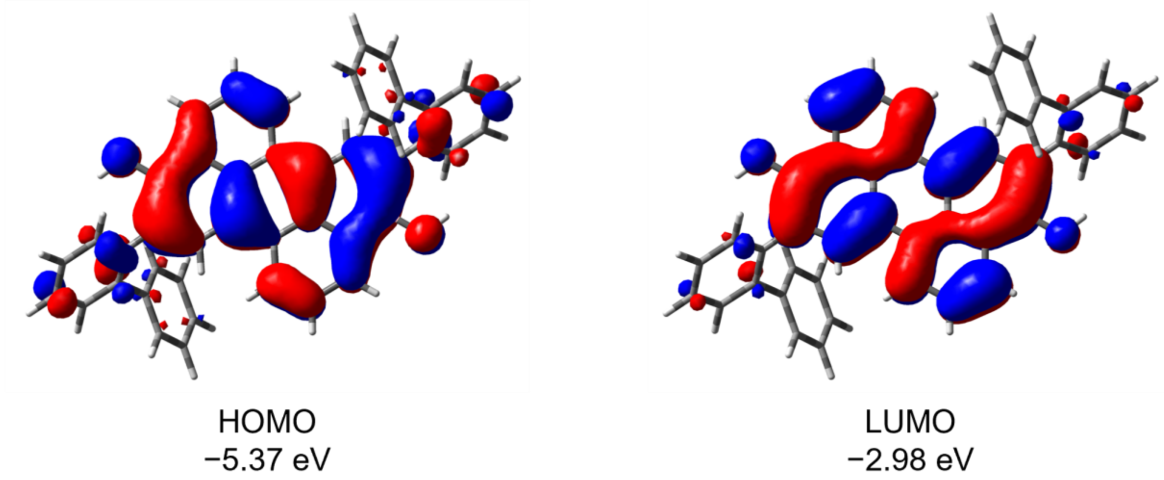

Figure S21. Frontier molecular orbitals of **2** by DFT calculations at the B3LYP/6-311G** level of theory. Isovalues for orbitals: 0.02 a.u.

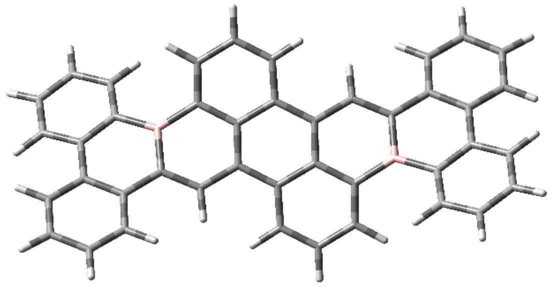

Figure S22. Geometry-optimized structure of **B_2-HBP** by DFT calculations at the B3LYP/6-311G** level of theory.

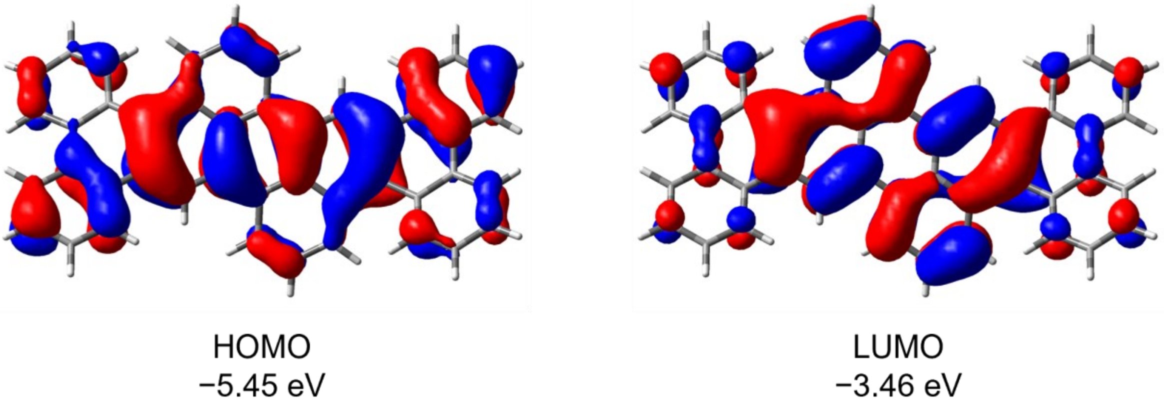

Figure S23. Frontier molecular orbitals of **B$_2$-HBP** by DFT calculations at the B3LYP/6-311G** level of theory. Isovalues for orbitals: 0.02 a.u.

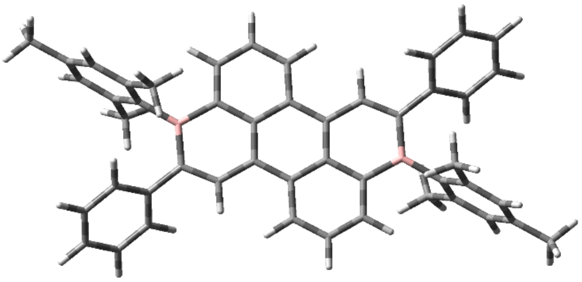

Figure S24. Geometry-optimized structure of **III** by DFT calculations at the B3LYP/6-311G** level of theory.

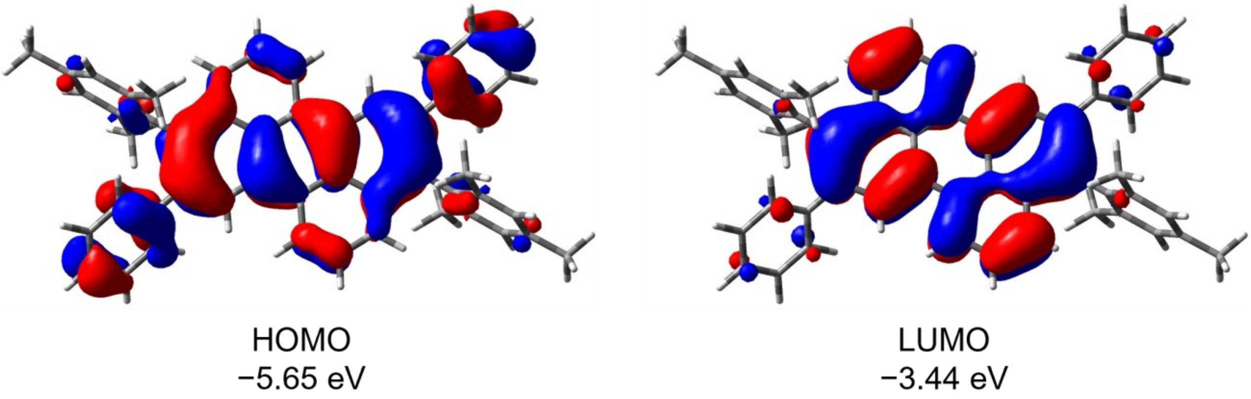

Figure S25. Frontier molecular orbitals of **III** by DFT calculations at the B3LYP/6-311G** level of theory. Isovalues for orbitals: 0.02 a.u.

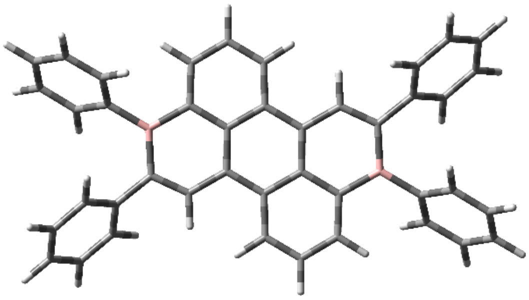

Figure S26. Geometry-optimized structure of **Ph-III** by DFT calculations at the B3LYP/6-311G** level of theory.

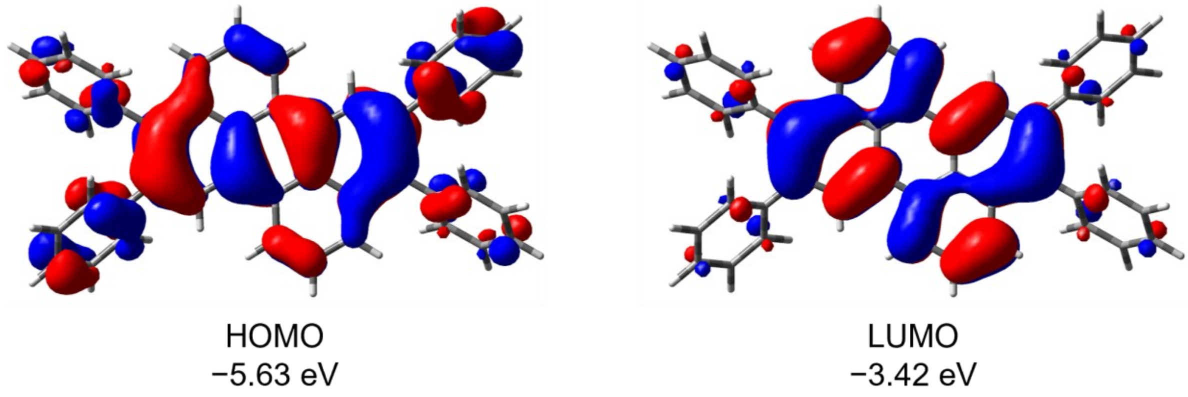

Figure S27. Frontier molecular orbitals of **Ph-III** by DFT calculations at the B3LYP/6-311G** level of theory. Isovalues for orbitals: 0.02 a.u.

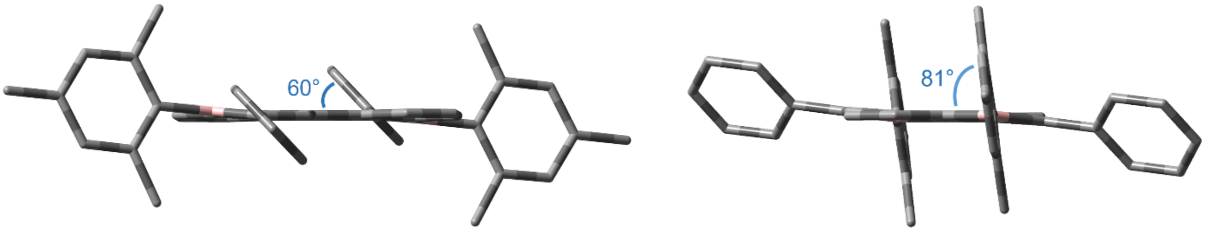

Figure S28. Side view of geometry-optimized structures and corresponding core-aryl substituents angles by DFT calculations at the B3LYP/6-311G** level of theory of **III**.

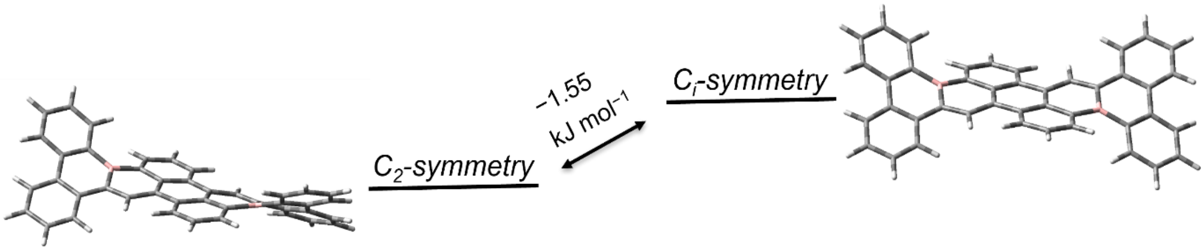

Figure S29. Side view of C_2- (left) and C_i- symmetric (right) geometry-optimized structures for **B_2-HBP**. The corresponding dihedral angle of both structures is 35°. DFT calculations were performed at the B3LYP/6-311G** level of theory.

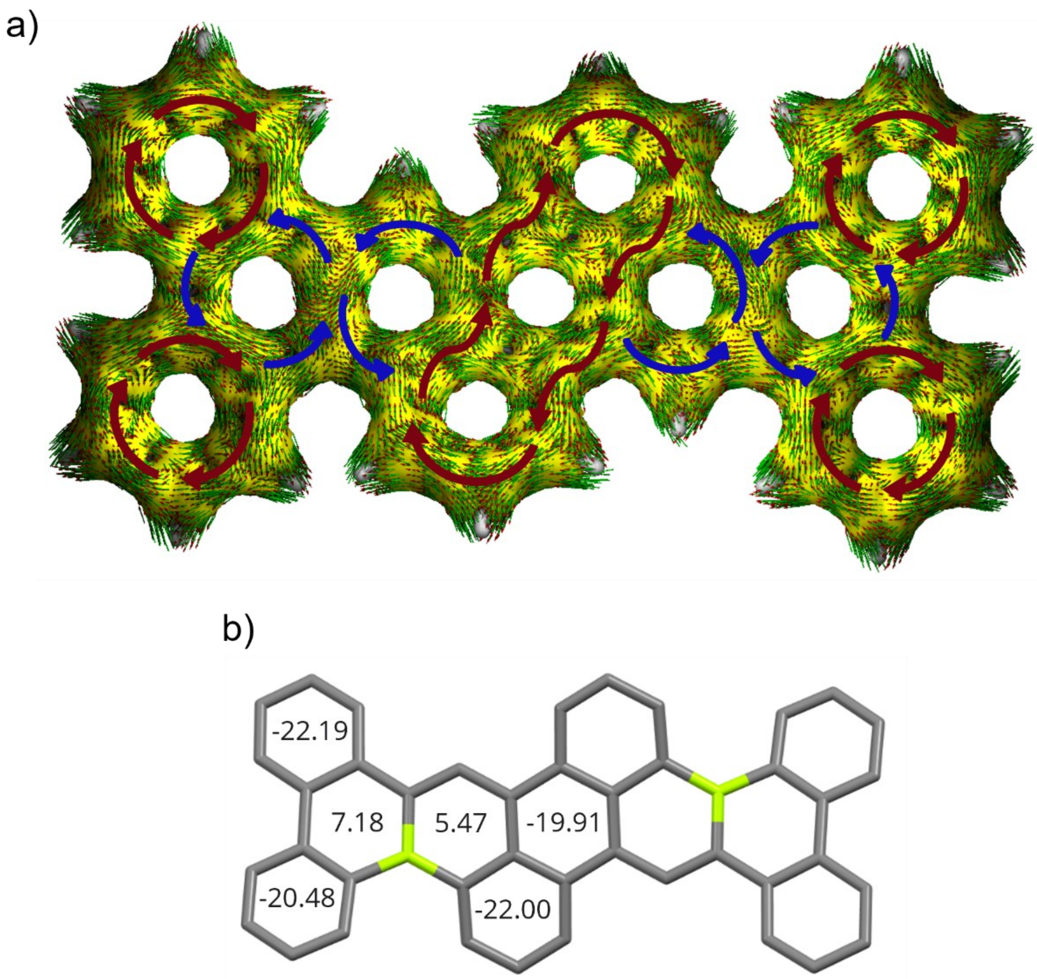

Figure S30. a) Calculated AICD plot (isovalue: 0.05) of **B_2-HBP**. Arrows in red represent clockwise ring current (aromaticity), arrows in blue counterclockwise ring current (antiaromaticity). b) NICS(1)$_{zz}$ values of B_2-HBP. All calculations were performed at the B3LYP/6-311G** level of theory.

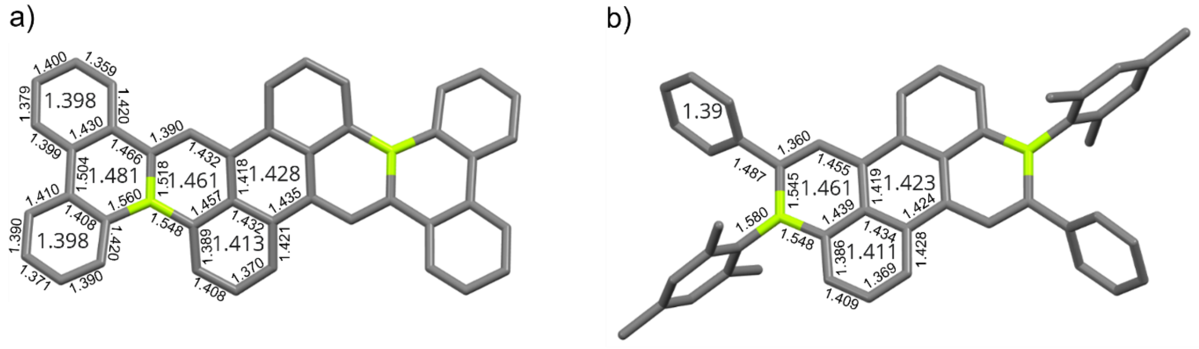

B_2-HBP **III**

Figure S31. Visualization of C−C and B−C bond lengths of the solid-state structure of **B_2-HBP** (a) and **III** (b) (all values in Å). The values in each ring correspond to the average bonds lengths of the respective ring.

Table S3. Absorption properties of compounds **III** and **B_2-HBP** as obtained from TD-DFT calculations (B3LYP/6- 311G**). The relevant transitions are listed with the respective wavelengths and oscillator strengths as well as the orbitals that are mainly involved in the transitions

Compound	Wavelength [nm]	Oscillator strength	Character
**III**	642	0.5093	HOMO → LUMO (69%)
	396	0.1658	HOMO−9 → LUMO (64%)
**B_2-HBP**	701	0.5887	HOMO → LUMO (69%)
	569	0.1396	HOMO−2 → LUMO (69%)
	402	0.1494	HOMO−6 → LUMO (66%)

Table S4. Cartesian coordinates and absolute energy of the geometry-optimized structure (B3LYP/6-311G**) of **2**.

Atoms	X	Y	Z
C	0.47525100	-3.09833284	2.03700582
C	-0.90830516	-3.15752382	1.77771283
C	-1.53856970	-2.18440962	1.02742677
C	-0.77239062	-1.08493733	0.50752453
C	0.63944659	-1.00936058	0.77311723
C	1.22115661	-2.05810930	1.54779274
C	-1.41329654	-0.08066073	-0.26687194
C	-0.63952509	1.00903349	-0.77238656
C	0.77230688	1.08463810	-0.50675133
C	1.41322796	0.08030265	0.26756152
C	-1.22124267	2.05781452	-1.54701306
C	-0.47536553	3.09811776	-2.03610088
C	0.90816967	3.15737001	-1.77671353
C	1.53844986	2.18421308	-1.02649963
B	3.05824321	2.27566334	-0.73562160
C	3.67400780	1.15193786	0.13235043
C	2.83640616	0.16529583	0.55086773
B	-3.05838793	-2.27581788	0.73664033
C	-3.67410806	-1.15226983	-0.13160859
C	-2.83644569	-0.16573216	-0.55027949
C	5.61745108	2.30163033	1.15184072
C	6.93830591	2.38757858	1.57898142
C	7.79961618	1.31670772	1.36281025
C	7.32655389	0.17887805	0.71850008
C	5.99932735	0.07385969	0.27795209
C	5.11332173	1.15467248	0.51142588
C	-5.11337955	-1.15506165	-0.51079946
C	-5.61765857	-2.30243201	-1.15036600
C	-6.93849049	-2.38849930	-1.57755912
C	-7.79964910	-1.31732642	-1.36230564
C	-7.32644711	-0.17906664	-0.71885026
C	-5.99924924	-0.07392082	-0.27826809
C	-5.60091615	1.17099079	0.44208697
C	5.60108173	-1.17056025	-0.44330181
C	5.82956247	-2.42521946	0.13773855
C	5.50684642	-3.59837338	-0.54127726
C	4.95497621	-3.53775361	-1.81807791
C	4.73015652	-2.29607451	-2.41076402
C	5.04717914	-1.12525379	-1.72976625
C	-5.04736449	1.12659271	1.72873086
C	-4.73027539	2.29789187	2.40887795
C	-4.95466482	3.53914426	1.81513770
C	-5.50619363	3.59886353	0.53814642
C	-5.82898434	2.42523597	-0.14001472
O	3.77703393	3.31610213	-1.26348035
O	-3.77720107	-3.31608834	1.26480250
H	0.95311099	-3.87694067	2.62119424
H	-1.49537121	-3.98208307	2.16757262
H	2.28037424	-2.05004518	1.75742264
H	-2.28045246	2.04973759	-1.75667473

H	-0.95323378	3.87674521	-2.62025626
H	1.49520453	3.98201947	-2.16642994
H	3.25957806	-0.62498283	1.15697051
H	-3.25953995	0.62438531	-1.15663673
H	4.93802538	3.12167452	1.35965542
H	7.28701552	3.28187675	2.08315942
H	8.83379961	1.36771892	1.68385855
H	8.00345362	-0.64531244	0.52306027
H	-4.93836365	-3.12275187	-1.35751872
H	-7.28728518	-3.28313547	-2.08107820
H	-8.83381660	-1.36842060	-1.68339061
H	-8.00322401	0.64539152	-0.52411489
H	6.25217337	-2.47913552	1.13527278
H	5.68484281	-4.55878471	-0.07011664
H	4.70253111	-4.44908621	-2.34816858
H	4.30778570	-2.23875147	-3.40775321
H	4.87430910	-0.16575081	-2.20261531
H	-4.87480182	0.16742967	2.20238299
H	-4.30818628	2.24127752	3.40602718
H	-4.70215214	4.45084456	2.34456295
H	-5.68386992	4.55893680	0.06617688
H	-6.25134124	2.47843937	-1.13769509
H	4.71182466	3.27514199	-1.03278386
H	-4.71200532	-3.27512337	1.03416772

E = −1818.09199720 a.u.

Table S5. Cartesian coordinates and absolute energy of the geometry-optimized C_i-structure (B3LYP/6-311G**) of **B_2-HBP**.

Atoms	X	Y	Z
C	-2.34587087	-2.68818500	-1.09508352
C	-1.10369486	-3.28010069	-1.39547170
C	0.06064423	-2.63015212	-1.07871257
C	0.05506808	-1.33013850	-0.49473084
C	-1.21611339	-0.70373159	-0.27332498
C	-2.43638206	-1.42944164	-0.52108987
C	1.27280777	-0.64767150	-0.17195992
C	1.21611033	0.70357128	0.27355773
C	-0.05507048	1.32995251	0.49500750
C	-1.27279749	0.64753628	0.17210464
C	2.43636147	1.42929940	0.52137016
C	2.34582397	2.68796261	1.09553890
C	1.10363930	3.27980010	1.39604896
C	-0.06068593	2.62987534	1.07918824
C	-2.53182688	1.35046058	0.19758627
C	2.53187893	-1.35051155	-0.19764935
C	-3.74808433	0.78121547	-0.07099702
C	3.74812779	-0.78123435	0.07090415
C	-4.99338155	1.55061145	-0.17239242
C	-4.98361419	2.93874354	-0.40160055
C	-6.15223354	3.66144470	-0.57530907
C	-7.37757256	2.99613685	-0.54449276
C	-7.41179181	1.62684342	-0.32634896
C	-6.24298299	0.87307980	-0.12080085
C	4.99346514	-1.55058367	0.17210463
C	4.98378716	-2.93873407	0.40120548
C	6.15245955	-3.66139309	0.57474031
C	7.37776370	-2.99602441	0.54387227
C	7.41189531	-1.62670777	0.32585281
C	6.24303051	-0.87298961	0.12046414
B	3.79424590	0.74480458	0.20538281
B	-3.79426354	-0.74483945	-0.20528993
C	5.16091826	1.41220465	-0.08586652
C	-5.16098435	-1.41213014	0.08598223
C	-6.32039643	-0.58532732	0.15683303
C	-7.54264813	-1.17205961	0.52715051
C	-7.64320276	-2.52704265	0.81281768
C	-6.51287259	-3.34101941	0.75863970
C	-5.29446463	-2.77723999	0.40983532
C	6.32034791	0.58545111	-0.15699876
C	7.54253256	1.17226829	-0.52740730
C	7.64302067	2.52730762	-0.81282585
C	6.51268093	3.34125230	-0.75832014
C	5.29432789	2.77737964	-0.40947819
H	-3.25112626	-3.22556473	-1.35017965
H	-1.06846335	-4.25268160	-1.87332748
H	0.99915360	-3.10827559	-1.31969853
H	3.25107163	3.22533035	1.35069178
H	1.06839451	4.25229293	1.87408248
H	-0.99921964	3.10791135	1.32026080

H	−2.48975296	2.40832637	0.42447130
H	2.48987746	−2.40834350	−0.42469513
H	−4.03395403	3.45382760	−0.48442503
H	−6.11023538	4.72888941	−0.76043418
H	−8.30159705	3.53949012	−0.70659920
H	−8.37397009	1.13255360	−0.33917668
H	4.03415829	−3.45386770	0.48408596
H	6.11052918	−4.72885502	0.75978229
H	8.30182804	−3.53934622	0.70585655
H	8.37404290	−1.13235677	0.33866714
H	−8.43131258	−0.56346849	0.62899957
H	−8.60217719	−2.94268467	1.10286138
H	−6.58079747	−4.39367170	1.00937082
H	−4.41033658	−3.40241180	0.42519468
H	8.43117441	0.56368988	−0.62954343
H	8.60194234	2.94302722	−1.10293242
H	6.58056034	4.39396517	−1.00880938
H	4.41017995	3.40252846	−0.42462924

E = −1665.10444258 a.u.

Table S6. Cartesian coordinates and absolute energy of the geometry-optimized *C*$_2$-structure (B3LYP/6-311G**) of **B$_2$-HBP**.

Atoms	X	Y	Z
C	-2.33519356	2.90856834	-0.01057913
C	-1.09443361	3.56878278	-0.09754425
C	0.06803956	2.84417149	-0.13723699
C	0.06143026	1.41896784	-0.13436174
C	-1.21131765	0.75874075	-0.07916885
C	-2.42609151	1.52587859	0.03053399
C	1.27646961	0.66076629	-0.17455661
C	1.21130463	-0.75870928	-0.07910900
C	-0.06144135	-1.41893949	-0.13430464
C	-1.27647403	-0.66073873	-0.17457043
C	2.42608197	-1.52583966	0.03058778
C	2.33519156	-2.90853111	-0.01053457
C	1.09443017	-3.56874921	-0.09750338
C	-0.06804891	-2.84414609	-0.13714819
C	-2.54639878	-1.31515698	-0.37343314
C	2.54639625	1.31518536	-0.37339853
C	-3.76088098	-0.68244047	-0.35752440
C	3.76088463	0.68247661	-0.35747253
C	-5.02280493	-1.35006776	-0.69658927
C	-5.04555180	-2.54501621	-1.43889523
C	-6.23192816	-3.14258953	-1.83068955
C	-7.44344745	-2.53509393	-1.50192217
C	-7.44596803	-1.35301764	-0.77642026
C	-6.25784747	-0.73949807	-0.34221574
C	5.02281530	1.35009383	-0.69652011
C	5.04558364	2.54512779	-1.43868597
C	6.23196102	3.14269430	-1.83047727
C	7.44347423	2.53510289	-1.50185366
C	7.44597650	1.35295585	-0.77647175
C	6.25785186	0.73943665	-0.34226721
B	3.78417329	-0.77630368	0.11192546
B	-3.78419329	0.77635993	0.11184253
C	5.12787199	-1.28353299	0.69016910
C	-5.12790070	1.28352993	0.69013183
C	-6.30019430	0.50088575	0.47634992
C	-7.49969736	0.90796323	1.08541019
C	-7.56510435	2.04724241	1.87625512
C	-6.42064589	2.80970417	2.10434655
C	-5.22443398	2.41651633	1.52273495
C	6.30020478	-0.50098497	0.47624731
C	7.49973810	-0.90816208	1.08518961
C	7.56512033	-2.04741613	1.87607031
C	6.42061123	-2.80974896	2.10434085
C	5.22437330	-2.41646642	1.52285057
H	-3.23947956	3.50450424	0.00919346
H	-1.05788591	4.65221725	-0.11737783
H	1.00550563	3.38040877	-0.15857829
H	3.23948559	-3.50445695	0.00919993
H	1.05788789	-4.65218386	-0.11732362
H	-1.00551954	-3.38037874	-0.15837312

H	−2.51814257	−2.38107885	−0.56024387
H	2.51812894	2.38110277	−0.56023162
H	−4.10842147	−2.99212625	−1.74858784
H	−6.21513694	−4.05842780	−2.41072762
H	−8.38191436	−2.97188184	−1.82418114
H	−8.39932985	−0.88842955	−0.56319627
H	4.10846398	2.99234697	−1.74825890
H	6.21517287	4.05861653	−2.41038273
H	8.38194209	2.97187474	−1.82413030
H	8.39932731	0.88830312	−0.56334409
H	−8.39739946	0.31459468	0.97401246
H	−8.50723269	2.32532420	2.33625910
H	−6.46037117	3.68286760	2.74587186
H	−4.32760516	2.97988633	1.74856228
H	8.39749244	−0.31489517	0.97367955
H	8.50727280	−2.32556607	2.33598335
H	6.46031862	−3.68286359	2.74593390
H	4.32750456	−2.97971285	1.74882036

E = −1665.10424743 a.u.

Table S7. Cartesian coordinates and absolute energy of the geometry-optimized structure (B3LYP/6-311G**) of **III**.

Atoms	X	Y	Z
C	1.60400022	-3.31759520	0.60602923
C	2.73616148	-2.49620861	0.44551379
C	2.62265066	-1.13709428	0.19481741
C	1.30648081	-0.56375835	0.10435849
C	0.14240838	-1.39386565	0.23606932
C	0.34720339	-2.78098217	0.49769736
C	1.16817316	0.83126425	-0.11538199
C	-0.14236430	1.39385396	-0.23625269
C	-1.30644111	0.56376540	-0.10448243
C	-1.16814010	-0.83124017	0.11539051
C	-0.34712541	2.78091582	-0.49817936
C	-1.60391260	3.31751262	-0.60669789
C	-2.73608789	2.49617144	-0.44603788
C	-2.62260122	1.13710681	-0.19505387
B	-3.90021040	0.26409025	-0.03741508
C	-3.64483497	-1.25863159	0.09344130
C	-2.34195019	-1.67114043	0.18379246
B	3.90025042	-0.26402892	0.03734147
C	3.64484149	1.25872358	-0.09312314
C	2.34195663	1.67122576	-0.18345361
C	4.70749415	2.29828031	-0.10689268
C	-4.70749326	-2.29816412	0.10762252
C	-4.55734191	-3.48453765	-0.63268555
C	-5.53372090	-4.47584127	-0.61524146
C	-6.68892844	-4.30731515	0.14517301
C	-6.85891260	-3.13483837	0.87864518
C	-5.88611771	-2.14009086	0.85453313
C	4.55726953	3.48442015	0.63376859
C	5.53364306	4.47573856	0.61671313
C	6.68891261	4.30745846	-0.14365905
C	6.85896690	3.13520820	-0.87748057
C	5.88618068	2.14044430	-0.85375484
C	-5.33035316	0.93605830	-0.02955475
C	-5.82163010	1.57778506	1.12838567
C	-7.08577967	2.17244761	1.11454541
C	-7.88966231	2.16494578	-0.02514027
C	-7.39305073	1.53839883	-1.16853299
C	-6.13972987	0.92383977	-1.18665904
C	-5.65662872	0.25727870	-2.45624658
C	-4.99935274	1.62229037	2.40021945
C	-9.26488967	2.78871739	-0.01476547
C	5.33037777	-0.93602302	0.02923881
C	6.13974305	-0.92431512	1.18635385
C	7.39303126	-1.53894049	1.16799004
C	7.88960695	-2.16506790	0.02435643
C	7.08572956	-2.17206893	-1.11533981
C	5.82161910	-1.57733024	-1.12894922
C	4.99928504	-1.62137502	-2.40076034
C	5.65660168	-0.25834723	2.45623563

C	9.26478633	−2.78894592	0.01373179
H	1.72485449	−4.37485652	0.81361063
H	3.72506324	−2.93495747	0.52298718
H	−0.50062446	−3.43757138	0.62672588
H	0.50072582	3.43745155	−0.62735129
H	−1.72475040	4.37472464	−0.81453866
H	−3.72497968	2.93491823	−0.52364223
H	−2.17145771	−2.73165066	0.32207105
H	2.17142738	2.73178125	−0.32136668
H	−3.67595090	−3.61521129	−1.25108400
H	−5.39621440	−5.37621780	−1.20415074
H	−7.45191371	−5.07747136	0.15927049
H	−7.75479937	−2.99120976	1.47260458
H	−6.03772064	−1.23457619	1.42659449
H	3.67582266	3.61490441	1.25212745
H	5.39607814	5.37592871	1.20589356
H	7.45189588	5.07762226	−0.15745515
H	7.75490330	2.99177251	−1.47141197
H	6.03783647	1.23510695	−1.42608246
H	−7.45009863	2.65506023	2.01743737
H	−7.99814295	1.52437280	−2.07101421
H	−6.39133487	0.35535486	−3.25811697
H	−4.71748793	0.69528452	−2.80987774
H	−5.47609397	−0.81051786	−2.29823696
H	−4.65861439	0.62530862	2.69880391
H	−4.10272840	2.23804922	2.27993627
H	−5.57787122	2.03603529	3.22883868
H	−9.33731017	3.58279633	0.73222705
H	−9.51663771	3.21583202	−0.98876128
H	−10.03390656	2.04502767	0.22268071
H	7.99813364	−1.52527705	2.07047012
H	7.45003688	−2.65431751	−2.01843121
H	5.57788021	−2.03446431	−3.22965394
H	4.10287628	−2.23750605	−2.28075166
H	4.65818860	−0.62434405	−2.69876516
H	4.71805105	−0.69732457	2.81024564
H	6.39177340	−0.35580628	3.25775399
H	5.47494635	0.80928771	2.29844823
H	9.51660638	−3.21622554	0.98763608
H	9.33704596	−3.58292618	−0.73338206
H	10.03384507	−2.04529662	−0.22370342

E = −1903.44564591 a.u.

Table S8. Cartesian coordinates and absolute energy of the geometry-optimized structure (B3LYP/6-311G**) of **Ph-III**.

Atoms	X	Y	Z
C	-1.19945818	3.50852464	-0.43644345
C	-2.42209039	2.81697493	-0.34684174
C	-2.47572289	1.44450266	-0.15011580
C	-1.23418237	0.71724938	-0.07736901
C	0.02072797	1.41215117	-0.14797281
C	-0.01609753	2.82639864	-0.32863714
C	-1.25631216	-0.69548241	0.05346069
C	-0.02077550	-1.41223282	0.14775475
C	1.23413561	-0.71732526	0.07719146
C	1.25625854	0.69540964	-0.05359435
C	0.01606239	-2.82649558	0.32830237
C	1.19942344	-3.50862924	0.43605096
C	2.42205350	-2.81706414	0.34654150
C	2.47568063	-1.44457457	0.14992783
B	3.84415707	-0.71020000	0.05656764
C	3.76266720	0.83410377	-0.00797224
C	2.51840007	1.39879633	-0.07387866
B	-3.84420849	0.71016899	-0.05674284
C	-3.76274755	-0.83413220	0.00813445
C	-2.51846990	-1.39882726	0.07397812
C	5.20012706	-1.50400113	0.04185535
C	5.45061584	-2.53205624	-0.88723503
C	6.65756516	-3.22857990	-0.89884090
C	7.64088148	-2.93476976	0.04296250
C	7.41559019	-1.92846776	0.98120011
C	6.22132450	-1.21408997	0.96659758
C	4.93572961	1.74899302	-0.03234964
C	-4.93578346	-1.74903404	0.03292070
C	-5.20012466	1.50404079	-0.04229450
C	5.99820046	1.55002953	-0.92777600
C	7.06925156	2.43652393	-0.97527273
C	7.11514600	3.53379559	-0.11669236
C	6.07618680	3.73902470	0.78791775
C	4.99820076	2.85829252	0.82667937
C	-5.99824391	-1.54973270	0.92828793
C	-7.06924527	-2.43626583	0.97621576
C	-7.11510832	-3.53392401	0.11813191
C	-6.07616775	-3.73949439	-0.78642599
C	-4.99823499	-2.85872176	-0.82561755
C	-5.45058753	2.53237500	0.88650201
C	-6.65746186	3.22902846	0.89782028
C	-7.64072076	2.93508760	-0.04400120
C	-7.41544795	1.92853106	-0.98197308
C	-6.22126884	1.21402491	-0.96707888
H	-1.19345956	4.58137362	-0.59273521
H	-3.34644644	3.37366228	-0.44179935
H	0.90397879	3.38702405	-0.40138029
H	-0.90400563	-3.38713438	0.40101857
H	1.19342140	-4.58148973	0.59226378
H	3.34640919	-3.37375444	0.44144724

H	2.47461288	2.47685868	−0.16653828
H	−2.47469983	−2.47686732	0.16686374
H	4.69508151	−2.77930282	−1.62632986
H	6.82749424	−4.00404793	−1.63818881
H	8.57710530	−3.48222159	0.04379196
H	8.17704270	−1.69168124	1.71646648
H	6.07615632	−0.41902679	1.69002689
H	5.97523433	0.70072213	−1.59930478
H	7.87256110	2.26820172	−1.68410196
H	7.95503274	4.21869383	−0.14930358
H	6.10598964	4.58286405	1.46870877
H	4.20211919	3.01559465	1.54659906
H	−5.97530360	−0.70013136	1.59944232
H	−7.87253977	−2.26766415	1.68499568
H	−7.95495622	−4.21885370	0.15107521
H	−6.10594651	−4.58363725	−1.46684165
H	−4.20218679	−3.01629056	−1.54551580
H	−4.69507854	2.77973589	1.62558633
H	−6.82737761	4.00470256	1.63695472
H	−8.57688178	3.48264675	−0.04506506
H	−8.17685071	1.69166438	−1.71726479
H	−6.07611155	0.41876972	−1.69029745

E = −1667.49084321 a.u.

8) X-ray crystallography

Crystal data for **B₂-HBP** ($C_{42}H_{24}B_2$): *Mr* = 550.23, 0.100 × 0.005 × 0.005 mm^3, monoclinic space group *C*2/*c*, a = 19.83(3) Å, α = 90°, b = 3.975(7) Å, β = 106.14(3)°, c = 33.65(5) Å, γ = 90°, V = 2548(7) Å^3, Z = 4, $\rho(calcd)$ = 1.435 g·cm^{-3}, μ = 0.062 mm^{-1} , $F_{(000)}$ = 1144, GooF(F^2) = 0.997, R_1 = 0.0989, wR_2 = 0.2502 for $I > 2\sigma(I)$, R_1 = 0.1815, wR_2 = 0.3361 for all data, 2180 unique reflections [$\theta \leq$ 21.647°] with a completeness of 96.8% and 199 parameters, 0 restraints.

9) References

[1] M. M. Brahmi, J. Monot, M. Desage-El Murr, D. P. Curran, L. Fensterbank, E. Lacôte, M. Malacria, *J. Org. Chem.* **2010**, *75*, 6983–6985.

[2] J. L. Nallasivam, R. A. Fernandes, *Eur. J. Org. Chem.* **2015**, *2015*, 3558–3567.

[3] Y. N. Xue, S. Y. Chai, G. J. Bie, B. Liu, N. Gan, *Acta Chim. Sinica* **2008**, *66*, 1577–1582.

[4] K. Rurack, M. Spieles, *Anal. Chem.* **2011**, *83*, 1232–1242.

[5] A. J. Fry, *in Laboratory Techniques in Electroanalytical Chemistry*, 2 ed., Marcel Dekker, New York, **1996**.

[6] W. Kabsch, *Acta Crystallogr. D* **2010**, *66*, 125−132.

[7] G. M. Sheldrick, *Acta Crystallogr. A* **2015**, *71*, 3–8.

[8] G. M. Sheldrick, *Acta Crystallogr. C* **2015**, *71*, 3–8.

[9] a) A. D. Becke, *Phys. Rev. A* **1988**, *38*, 3098–3100; b) C. Lee, W. Yang, R. G. Parr, *Phys. Rev. B* **1988**, *37*, 785–789; c) A. D. Becke, *J. Chem. Phys.* **1993**, *98*, 1372–1377.

[10] a) R. Krishnan, J. S. Binkley, R. Seeger, J. A. Pople, *J. Chem. Phys.* **1980**, *72*, 650–654; b) T. Clark, J. Chandrasekhar, G. W. Spitznagel, P. V. R. Schleyer, *J. Comput. Chem.* **1983**, *4*, 294–301; c) M. J. Frisch, J. A. Pople, J. S. Binkley, *J. Chem. Phys.* **1984**, *80*, 3265–3269.

[11] M. J. Frisch, G. W. Trucks, H. B. Schlegel, G. E. Scuseria, M. A. Robb, J. R. Cheeseman, G. Scalmani, V. Barone, G. A. Petersson, H. Nakatsuji, X. Li, M. Caricato, A. V. Marenich, J. Bloino, B. G. Janesko, R. Gomperts, B. Mennucci, H. P. Hratchian, J. V. Ortiz, A. F. Izmaylov, J. L. Sonnenberg, Williams, F. Ding, F. Lipparini, F. Egidi, J. Goings, B. Peng, A. Petrone, T. Henderson, D. Ranasinghe, V. G. Zakrzewski, J. Gao, N. Rega, G. Zheng, W. Liang, M. Hada, M. Ehara, K. Toyota, R. Fukuda, J. Hasegawa, M. Ishida, T. Nakajima, Y. Honda, O. Kitao, H. Nakai, T. Vreven, K. Throssell, J. A. Montgomery Jr., J. E. Peralta, F. Ogliaro, M. J. Bearpark, J. J. Heyd, E. N. Brothers, K. N. Kudin, V. N. Staroverov, T. A. Keith, R. Kobayashi, J. Normand, K. Raghavachari, A. P. Rendell, J. C. Burant, S. S. Iyengar, J. Tomasi, M. Cossi, J. M. Millam, M. Klene, C. Adamo, R. Cammi, J. W. Ochterski, R. L. Martin, K. Morokuma, O. Farkas, J. B. Foresman, D. J. Fox, Wallingford, CT, **2013**.

[12] R. Dennington, T. Keith, J. Millam, Semichem Inc., **2009**.
